# Motion-invariant variational autoencoding of brain structural connectomes

**DOI:** 10.1162/imag_a_00303

**Published:** 2024-10-07

**Authors:** Yizi Zhang, Meimei Liu, Zhengwu Zhang, David Dunson

**Affiliations:** Department of Statistics, Columbia University, New York, NY, United States; Department of Statistics, Virginia Polytechnic Institute and State University, Blacksburg, VA, United States; Department of Statistics and Operations Research, The University of North Carolina at Chapel Hill, Chapel Hill, NC, United States; Department of Statistical Science, Duke University, Durham, NC, United States

**Keywords:** brain structural connectomes, motion correction, diffusion imaging, graph neural networks, invariant representations, variational autoencoders

## Abstract

Mapping of human brain structural connectomes via diffusion magnetic resonance imaging (dMRI) offers a unique opportunity to understand brain structural connectivity and relate it to various human traits, such as cognition. However, head displacement during image acquisition can compromise the accuracy of connectome reconstructions and subsequent inference results. We develop a generative model to learn low-dimensional representations of structural connectomes invariant to motion-induced artifacts, so that we can link brain networks and human traits more accurately, and generate motion-adjusted connectomes. We apply the proposed model to data from the Adolescent Brain Cognitive Development (ABCD) study and the Human Connectome Project (HCP) to investigate how our motion-invariant connectomes facilitate understanding of the brain network and its relationship with cognition. Empirical results demonstrate that the proposed motion-invariant variational autoencoder (inv-VAE) outperforms its competitors in various aspects. In particular, motion-adjusted structural connectomes are more strongly associated with a wide array of cognition-related traits than other approaches without motion adjustment.

## 1 Introduction

To comprehensively understand brain function and organization, enormous efforts have been dedicated to imaging and analyzing the brain structural connectome, defined as a collection of white matter fiber tracts connecting different brain regions. Recent advancements in noninvasive neuroimaging techniques have led to a rapid increase in brain imaging datasets, such as the Human Connectome Project (HCP) (Glasser et al., 2016) and the Adolescent Brain Cognitive Development (ABCD) study (Bjork et al., 2017). These developments have inspired a substantial body of literature (Zhang, Allen, et al., 2019;Zhang et al., 2018) that focuses on analyzing structural connectomes derived from diffusion-weighted magnetic resonance imaging (dMRI) data. However, these studies often encounter challenges due to subject head displacement during image acquisition, resulting in image misalignment and artifacts that hinder unbiased connectome reconstructions and analysis (Andersson et al., 2016). Addressing these biases is crucial, which has motivated us to develop novel statistical methods capable of modeling and analyzing brain structural connectomes in an invariant manner (Achille & Soatto, 2018;Zemel et al., 2013).

Our goal is to learn a low-dimensional representation of brain connectomes invariant to motion artifacts in the data, thereby facilitating prediction and inference on the relationship between brain structure and human traits such as cognitive abilities. A common motion correction technique in neuroimaging involves registering motion-corrupted images to a geometrically correct template (Bhushan et al., 2015;Kochunov et al., 2006). However, in dMRI, motion frequently causes geometric distortion and signal dropout, significantly complicating registration (Ben-Amitay et al., 2012) and impacting subsequent brain network analyses (Le Bihan et al., 2006). Current motion correction methods often utilize the FSL eddy tool (Andersson & Sotiropoulos, 2016) to estimate and correct for eddy current-induced distortions and subject movements. Recent advancements include techniques such as SHORELine (Cieslak et al., 2021) and SHARD (Christiaens et al., 2021) for correcting multishell dMRI sequences. Despite these improvements, studies have shown that even after motion correction in diffusion signals, in-scanner head motion can still have notable impacts on the recovered structural connectivity (Baum et al., 2018). Moreover, not all sources of image misalignment are due to motion (Yendiki et al., 2014). Various imperfections in the imaging system can lead to artifacts causing image misalignment. Given these challenges, our major motivation is to develop a method that addresses motion-induced artifacts and adjusts for residual misalignment during the brain network modeling process, rather than in the data preprocessing stage. Here, motion-induced artifacts refer to the in-scanner head displacement that can be approximately quantified using image registration algorithms (Andersson & Sotiropoulos, 2016). Residual misalignment encompasses some remaining artifacts measured after eddy motion correction. By incorporating these considerations into our modeling approach, we aim to improve the robustness and reliability of brain connectome analysis in the presence of motion-induced artifacts.

In biomedical imaging, removing unwanted effects in high-dimensional data has been an important problem for at least the last decade. Without proper adjustment, the association between the signal of interest and one or more nuisance variables can degrade the ability to infer the signal accurately. This issue is particularly evident in brain imaging studies, and various tools have been developed. A popular matrix factorization method (Alter et al., 2000) uses a singular value decomposition of the original matrix to filter out the singular vector associated with the nuisance variable, and reconstructs an adjusted matrix with the remaining singular vectors. Similarly, the Sparse Orthogonal to Group (SOG) (Aliverti et al., 2021) proposes an adjusted dataset based on a constrained form of matrix decomposition. In addition, model-based methods are popular alternatives for removing unwanted associations in the data. For example, distance-weighted discrimination (Benito et al., 2004) adapts Support Vector Machines (SVM) for batch effect removal; the ComBat method (Johnson et al., 2007) adjusts for batch effects in data using empirical Bayes. Our work falls under the model-based framework, and we are particularly interested in incorporating batch effect removal into a deep learning framework so that the nonlinear batch effect can be modeled and removed.

Removing unwanted information in the deep learning literature is formulated as an invariant representation learning problem. Existing deep learning methods learn invariant representations through either adversarial or nonadversarial methods. Adversarial learning methods, prevalent in domain adaptation, involve a generative adversarial network (GAN) (Creswell et al., 2018) where the generator extracts features to deceive the discriminator, while the discriminator aims to distinguish between different data domains. However, adversarial learning has limitations, including GANs’ mode collapse issues and sensitivity to hyperparameter choices (Papernot et al., 2016). Conversely, nonadversarial invariant methods, including recent group invariance techniques such as contrastive learning (Chen et al., 2020;He et al., 2020;Khosla et al., 2020) and conditionally invariant VAE (Aliee et al., 2023), aim to develop predictors invariant to a group’s action on the input space. Despite their strengths, these methods face a notable limitation: they are designed to handle categorical nuisance variables and may encounter challenges when confronted with continuous-valued nuisance variables. Some deep learning methods aim to mitigate the impact of continuous-valued nuisances, often incorporating mutual information loss. For instance,Greenfeld and Shalit (2020)utilize the Hilbert Schmidt Independence Criterion (HSIC), whileYu et al. (2021)replace HSIC with matrix-based mutual information, yielding performance improvements. Another variant, squared-loss MI (SMI) (Y. Liu et al., 2021), is also popular in this context. However, these mutual information-based methods focus solely on capturing dependencies in data in an unsupervised setting and do not ensure that the learned representations are task relevant. In our work, we advocate for learning motion-invariant representations guided by the information bottleneck principle (Alemi et al., 2016;Moyer et al., 2018). Our approach removes the mutual information between latent representations and undesirable artifacts, with latent representations learned via graph variational autoencoders (VAE) (M. Liu et al., 2021). Situated in the information bottleneck literature, our work addresses the challenge of finding representations that maximize the relationship with the target response while minimizing mutual information with nuisance variables such as subject motion and residual image misalignment. This framework not only addresses the noted limitations of existing methods but also correlates learned invariant representations with human behavior and cognition-related attributes.

We aim to model brain connectomes and learn their representations invariant to nuisance factors, particularly motion-related artifacts. Our analysis of ABCD and HCP data, preprocessed with FSL eddy software (Andersson & Sotiropoulos, 2016), reveals that structural connectome data are significantly affected by motion. This impact is observed not only in the motion quantified by FSL eddy but also in residual misalignment in the diffusion MRI data after FSL eddy correction. Notably, individuals exhibiting substantial head displacement during data acquisition tend to have fewer reconstructed fiber connections across nearly all brain regions. To address this issue, we develop a generative model that learns the distribution of brain connectomes while adjusting for undesirable artifacts induced by motion. This model also learns low-dimensional representations of structural connectomes that are invariant to motion. We achieve this by introducing a penalized VAE objective to eliminate mutual information between connectome representations and motion measures. The learned invariant representation enhances our understanding of motion’s impact on structural connectivity reconstructions. It can also be used to produce motion-adjusted connectomes for downstream analysis tasks, such as predicting behavior- and cognition-related traits and inferring structural connections between brain regions. Our analysis of ABCD and HCP data demonstrates that these motion-invariant representations improve the prediction of many cognitive traits by 5% to 25% compared with connectome representations without motion adjustment.

The rest of the paper is organized as follows:Section 2introduces the dMRI datasets used for deriving structural brain connectomes and outlines the motion measures utilized in this study. InSection 3, we present our method for adjusting for motion-induced artifacts during the modeling of structural brain networks.Section 4details a simulation study verifying the effectiveness of our method in removing unwanted information.Section 5applies our proposed method to two large neuroimaging studies, demonstrating its efficacy in correcting for motion-induced artifacts and establishing a more accurate analysis between structural connectomes and cognitive traits.Section 6includes a discussion. In theAppendix, we present additional results by considering the residual misalignment after FSL eddy processing as an alternative source of artifacts. Furthermore, we expand the simulation study to assess the robustness of our method when handling extreme motion-related artifacts in theAppendix.

## 2 Datasets Studied

### 2.1 Structural connectomes and cognitive traits

The ABCD study tracks the brain development of over 10,000 adolescents in the United States. We download and process the diffusion MRI (dMRI) data for 8,646 subjects from the NIH Data Archive. Details about data acquisition and preprocessing can be found inCasey et al. (2018). The Human Connectome Project (HCP) characterizes brain connectivity in more than 1,000 adults; seeVan Essen et al. (2012)for details about data acquisition. The latest dMRI data from the HCP can be accessed through ConnectomeDB. We download and process 1,065 subjects from the HCP.

To extract structural connectomes from the raw dMRI data, a reproducible tractography algorithm (Maier-Hein et al., 2017) is used to generate the whole-brain tractography for each subject. We use the Desikan–Killiany parcellation (Desikan et al., 2006) to define brain regions of interest (ROIs) corresponding to different nodes in the brain network. The Desikan–Killiany atlas contains 68 ROIs with 34 ROIs belonging to each hemisphere. We use Freesurfer (Iannopollo et al., 2019) to perform brain registration and parcellation. We extract streamlines connecting ROI pairs from the tractography data and the brain parcellation. Fiber count is often used to measure the coupling strength between ROI pairs in the current literature (Fornito et al., 2013;Zhang, Allen, et al., 2019), and, therefore, we summarize each brain connection with its fiber count.

ABCD and HCP use reliable measures to assess a wide range of human behaviors and functions (Gershon et al., 2013). We focus on inferring the relationship between structural connectomes and cognition-related traits, for example, scores from the picture vocabulary and oral reading recognition tests.

### 2.2 Motion quantification

Our study considers two sources of motion artifacts: head motion and residual misalignment. Head motion refers to the displacement of the head in the scanner estimated by the eddy tool (Andersson & Sotiropoulos, 2016). Residual misalignment, on the other hand, refers to any remaining misalignment after applying the eddy correction tool. While we analyze both types of artifacts, our primary focus is on head motion because we are interested in the impact of head motion on the diffusion-weighted images used for tractography and the subsequent construction of structural connectivity. We provide additional analysis of the residual misalignment inAppendix G. This section explores how head motion estimates are obtained and its impact on brain structural connectomes.

We primarily rely on the FSL eddy tool to estimate head motion. The eddy software corrects head motion in diffusion MRI data through a multistep process that involves modeling and correcting for both eddy currents and subject movement. It outputs a file named “my_eddy_output.eddy_movement_rms,” which provides summaries of the total movement in each volume. This is calculated by determining the displacement of each voxel, averaging the squares of those displacements across all intracerebral voxels, and finally taking the square root of that average. The file contains two columns: the first column lists the RMS (root mean square) movement relative to the first volume, and the second column lists the RMS movement relative to the previous volume. We use the second measure as our head motion metric in this paper (as it is the only head motion measure accessible in the ABCD data).

**Fig. 1.**
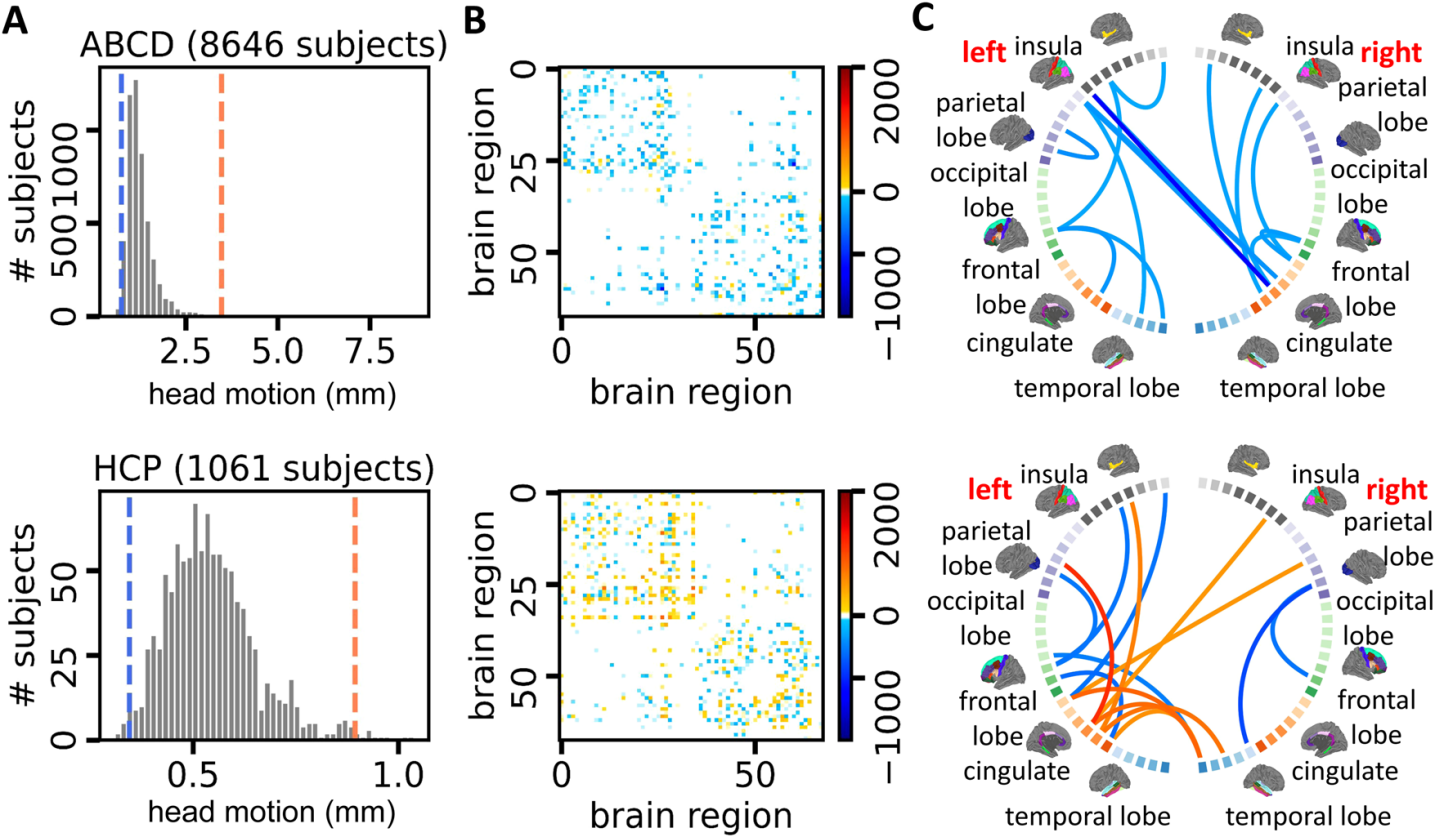
Head motion analysis for subjects in the ABCD and HCP datasets and the impact of motion on brain structural connectome data. (A) Distribution histograms of head motion for ABCD and HCP subjects, with the blue and orange lines representing the 10th and 90th percentiles. Subjects with head motion below the blue line are classified into the small motion group, while those with head motion above the orange line belong to the large motion group. (B) Mean network differences between the large and small motion groups (large–small). (C) Circular plots representing the data in panel (B). The color scale corresponds to that in (B), with the display limited to the 15 edges showing the largest fiber count differences.

Figure 1(A)shows distributions of head motion for the ABCD and HCP studies. We notice that the motion distribution of ABCD subjects is more right skewed with more subjects experiencing bigger motion during image acquisition. This disparity in motion measure distributions between the ABCD and HCP studies could be attributed to the demographic differences in their respective cohorts. Our hypothesis suggests that the HCP study focuses on adults, who tend to follow instructions better and exhibit less movement during image acquisition compared with adolescents in the ABCD study.

To explore the impact of head motion on structural connectomes, we assign individuals to a large and a small motion group corresponding to each movement type.Figure 1shows the cutoff lines for both ABCD and HCP data. Specifically, in the ABCD data, individuals whose motion estimates are ranked among the top (bottom) 5 percent are assigned to the large (small) motion group. In the HCP data, we use the top and the bottom 10 percent as the threshold. We then quantify brain connection differences between groups. We subtract edge-specific means of the small motion group from those of the large motion group and show the results as adjacency matrices inFigure 1(B). In addition, we visualize between-group differences using a circular layout inFigure 1(C)to examine how connection patterns and strength differ across ROIs. Note that the dMRI data used here are preprocessed with the FSL eddy tool and its motion correction procedures.Figure 1(B and C)reveals that brain networks of large-motion subjects show systematic differences in fiber counts across multiple ROIs compared with those of small-motion subjects, implying that motion artifacts still affect structural connection reconstruction even after motion correction in the preprocessing stage (Andersson & Sotiropoulos, 2016;Andersson et al., 2016). Motivated by these findings, we propose the motion-invariant graph variational autoencoder inSection 3to remove such systematic artifacts from the connectome data and downstream analyses.

## 3 Method

The brain connectome for an individual is represented as an undirected graphG=(V,ℰ)with nodesVand edgesℰ. The collection of nodesVrefers to the partitioned brain regions, and the set of edgesℰrepresents connections between pairs of ROIs. The ROIs are prealigned so that each node inVcorresponds to the same brain region across different subjects. We use the symmetric adjacency matrixAto representGwith$A_{uv}$denoting the number of streamlines connecting ROIsuandv;$A_{uu}=0$since we do not consider self-connections. ForNsubjects, we denote the network data as${\text{A}}_{i},i=1,2,\ldots ,N$. Also, denote the cognitive trait score for an individual as$y_{i}$, and the amount of motion as${\text{c}}_{i}$. Note that${\text{c}}_{i}$can be a scalar quantifying overall head displacement or a vector (${\text{c}}_{i}\in {\mathbb{R}}^{C}$) with each dimension measuring different aspects of motion such as the amplitudes of translation or rotation. Moreover,${\text{c}}_{i}$can represent other undesirable artifacts, such as the residual misalignment, depending on the research goal.

We aim to infer the relationship between individuals’ structural connectomes, recovered from neuroimaging data, and their measured cognitive traits, while adjusting for motion-induced artifacts during data acquisition. The key to achieving this goal is to learn a low-dimensional feature representation of${\text{A}}_{i}$as${\text{z}}_{i}$that is invariant to motion${\text{c}}_{i}$, and then relate this feature${\text{z}}_{i}$to$y_{i}$. Inspired by the variational autoencoder (VAE) framework inD. P. Kingma (2013), we achieve our goal in three steps: (1) an encoder module (inference model) that learns the mapping from the observation${\text{A}}_{i}$to the motion-invariant latent variable${\text{z}}_{i}$; (2) a decoder module (generative model) that specifies how to construct${\text{A}}_{i}$from${\text{z}}_{i}$and motion${\text{c}}_{\text{i}}$; and (3) a regression module to link the motion-invariant${\text{z}}_{i}$to the cognitive trait$y_{i}$; seeFigure 2for the model architecture. Intuitively, in the training process, our approach seeks to maximize the joint likelihood of$\left({\text{A}}_{i},y_{i}\right)$conditional on${\text{c}}_{i}$while minimizing the dependence between${\text{z}}_{i}$and${\text{c}}_{i}$. This is achieved by characterizing the joint likelihood through the brain network generative model inSection 3.1and addressing the dependence through mutual information between the low-dimensional representations of brain networks and motion, as detailed inSection 3.2.

**Fig. 2.**
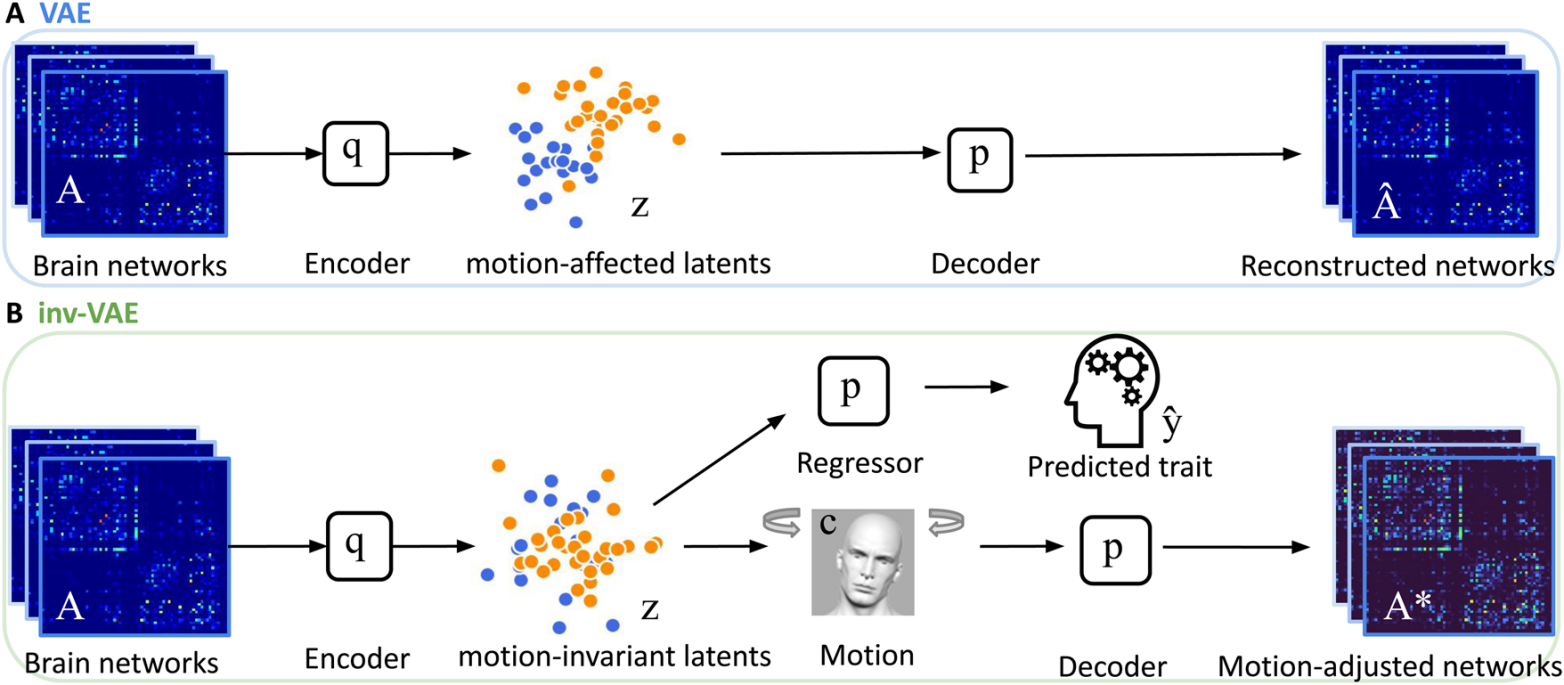
(A) Structure of the standard VAE model. The encoder learns motion-affected latents from motion-affected brain networks, and the decoder reconstructs networks from these latents. (B) Architecture of our proposed motion-invariant VAE. The encoder learns motion-invariant latents from the motion-affected networks, and the decoder reconstructs motion-adjusted networks from the invariant latents and the user-specified amount of subject motion. The invariant latents additionally serve as inputs to a regressor for predicting cognition-related traits.

In the following sections, we start with introducing our graph generative model and then discuss the encoder and regression modules.

### 3.1 Brain network generative model

For each individuali, we assume the edges$A_{i\left[uv\right]}$are conditionally independent given${\text{z}}_{i}$and${\text{c}}_{i}$. The likelihood of the set of edges in${\text{A}}_{i}$is

$$p_{\theta }\left({\text{A}}_{i}|{\text{z}}_{i},{\text{c}}_{i}\right)=\prod_{u=1}^{V}\prod_{v=1}^{V}p_{\theta }\left(A_{i\left[uv\right]}|{\text{z}}_{i},{\text{c}}_{i}\right),$$ (1)

where$p_{\theta }\left({\text{A}}_{i}|{\text{z}}_{i},{\text{c}}_{i}\right)$is the generative model for${\text{A}}_{i}$given${\text{z}}_{i}$and${\text{c}}_{i}$, parameterized byθlearned via neural networks. We assume${\text{A}}_{i}$to be generated from the following process:

$${\text{z}}_{i}∼N\left(\text{0},{\text{I}}_{K}\right),$$ (2)

$$A_{i\left[uv\right]}|\left({\text{z}}_{i},{\text{c}}_{i}\right)\;∼\text{Pois}\left(\lambda_{i\left[uv\right]}\left({\text{z}}_{i},{\text{c}}_{i}\right)\right),$$ (3)

whereKis the dimension of the latent feature space. The fiber count between ROIsuandvis modeled using a Poisson distribution with a parameter$\lambda_{i\left[uv\right]}\left({\text{z}}_{i},{\text{c}}_{i}\right)$, which relates to both the encoded feature${\text{z}}_{i}$and motion${\text{c}}_{i}$.

To capture the varying effect of motion across individuals and brain connections, we further model$\lambda_{i\left[uv\right]}\left({\text{z}}_{i},{\text{c}}_{i}\right)$as

$${\tilde{\text{z}}}_{i}:\;={\text{z}}_{i}⊕{\text{c}}_{i},$$ (4)

$$\lambda_{i\left[uv\right]}\left({\text{z}}_{i},{\text{c}}_{i}\right)=\text{exp}\left(\xi_{uv}+\psi_{uv}\left({\tilde{\text{z}}}_{i}\right)\right),$$ (5)

where⊕denotes the concatenation operator and${\tilde{\text{z}}}_{i}\in {\mathbb{R}}^{K+C}$is a concatenated vector representing motion-affected latent features.$\xi_{uv}$is a baseline parameter controlling the population connection strength between regionsuandv, representing shared structural similarity across individuals, and$\psi_{uv}\left({\tilde{\text{z}}}_{i}\right)$characterizes individual connection strength after accounting for motion.

We model$\psi_{uv}\left({\tilde{\text{z}}}_{i}\right)$based on the latent space model inHoff et al. (2002). The key idea is that the connection strength (fiber count) between regionsuandvdepends on their distance in some network latent space in${\mathbb{R}}^{R}$. In this network latent space, each brain region is represented by a vector in the${\mathbb{R}}^{R}$space, with the distance between two points reflecting the degree of association between the corresponding brain regions. The closer two points are in this space, the stronger their connection is expected to be. The network latent space is different from the latent space of${\tilde{\text{z}}}_{i}$, which represents a low-dimensional space where the original high-dimensional brain structural connectomes are encoded into a lower dimensional representation.

We construct a mappingXfrom${\mathbb{R}}^{K+C}$to${\mathbb{R}}^{V\times R}$that maps the motion-affected feature${\tilde{\text{z}}}_{i}$to the network latent space that captures the latent positions of all brain regions, that is,$\text{X}{\left({\tilde{\text{z}}}_{i}\right)}^{⊤}=\left({\text{X}}_{1}\left({\tilde{\text{z}}}_{i}\right),\ldots ,{\text{X}}_{V}\left({\tilde{\text{z}}}_{i}\right)\right)$with${\text{X}}_{u}\left({\tilde{\text{z}}}_{i}\right)={\left(X_{u1}\left({\tilde{\text{z}}}_{i}\right),...,X_{uR}\left({\tilde{\text{z}}}_{i}\right)\right)}^{⊤}\in {\mathbb{R}}^{R}$for brain regionu. Specifically, we assume each nodeu∈Vfor individualilies in a latent space${\mathbb{R}}^{R}$and represent$\psi_{uv}\left({\tilde{\text{z}}}_{i}\right)$as

$$\psi_{uv}\left({\tilde{\text{z}}}_{i}\right)=\sum_{r=1}^{R}\alpha_{r}X_{ur}\left({\tilde{\text{z}}}_{i}\right)X_{vr}\left({\tilde{\text{z}}}_{i}\right),$$ (6)

where$\alpha_{r}>0$is a weight controlling the importance of ther-th dimension. A large positive inner product between${\text{X}}_{u}\left({\tilde{\text{z}}}_{i}\right)$and${\text{X}}_{v}\left({\tilde{\text{z}}}_{i}\right)$implies a large fiber count between this ROI pair. Therefore,${\text{X}}_{u}\left({\tilde{\text{z}}}_{i}\right)$incorporates the geometric collaborations among nodes, and broadcasts the impact of motion to all brain connections.M. Liu et al. (2021)proposed a graph convolutional network (GCN) to learn a nonlinear mapping from the encoded feature${\text{z}}_{i}$to$\left({\text{X}}_{1}\left({\text{z}}_{i}\right),\ldots ,{\text{X}}_{V}\left({\text{z}}_{i}\right)\right),$leveraging on unique geometric features of the brain networks. The same procedure is adopted in this paper with the additional adjustment for motion${\text{c}}_{i}$, and we defer the detailed GCN framework toAppendix A.

### 3.2 Motion-invariant brain network encoding and traits prediction

We are interested in studying the relationship between brain structural networks and human traits, correcting for motion-related artifacts. The key is to find an optimal encoder that produces${\text{z}}_{i}$from${\text{A}}_{i}$invariant to${\text{c}}_{i}$, and then formulate a regression of the trait$y_{i}$with respect to the latent feature vector${\text{z}}_{i}$. We represent the inference model (encoder) as$q_{\phi }\left({\text{z}}_{i}|{\text{A}}_{i}\right)$, the generative model (decoder) as$p_{\theta }\left({\text{A}}_{i}|{\text{z}}_{i},{\text{c}}_{i}\right)$, and regression as$p_{\theta }\left(y_{i}|{\text{z}}_{i}\right)$. Our invariant encoding and trait prediction task is to find$q_{\phi }\left({\text{z}}_{i}|{\text{A}}_{i}\right)$and$p_{\theta }\left({\text{A}}_{i}|{\text{z}}_{i},{\text{c}}_{i}\right)$that maximize$E_{{\text{A}}_{i},y_{i},{\text{c}}_{i}}\left[\text{log}\;p_{\theta }\left({\text{A}}_{i},y_{i}|{\text{c}}_{i}\right)\right]$, subject to${\text{z}}_{i}$being independent of${\text{c}}_{i}$. Here$p_{\theta }\left({\text{A}}_{i},y_{i}|{\text{c}}_{i}\right)$is the joint likelihood of${\text{A}}_{i},y_{i}$conditional on${\text{c}}_{i}$. Since correlation is a limited notion of statistical dependence, which does not capture nonlinear dependencies, we instead use mutual information between${\text{z}}_{i}$and${\text{c}}_{i}$to quantify their dependency similar toMoyer et al. (2018), and formulate the objective function as follows:

$$ℒ\left({\text{A}}_{i},y_{i},{\text{c}}_{i};\theta ,\phi \right)=E_{{\text{A}}_{i},y_{i},{\text{c}}_{i}}\left[\text{log}\;p_{\theta }\left({\text{A}}_{i},y_{i}|{\text{c}}_{i}\right)\right]-\lambda I\left({\text{z}}_{i},{\text{c}}_{i}\right),$$ (7)

where$I\left({\text{z}}_{i},{\text{c}}_{i}\right)$is the mutual information between${\text{z}}_{i}$and${\text{c}}_{i}$, andλis a trade-off parameter between the joint likelihood and the mutual information.ϕare parameters of the encoder that are learned via neural networks.

To simplify our model, we assume (1) the human trait$y_{i}$and the brain connectivity${\text{A}}_{i}$are conditionally independent given the latent representation${\text{z}}_{i}$for individualiand (2) only${\text{A}}_{i}$is affected by${\text{c}}_{i}$so$y_{i}$is not affected. Assumption (2) indicates that motion happens randomly given the population considered and does not relate to the human traits (e.g., cognitive ability) considered. In certain scenarios, this assumption might not hold. For example, in the older population, people with mild cognitive impairment (MCI) tend to move more than normal controls (Haller et al., 2014), and if$y_{i}$is an indicator of MCI, Assumption (2) does not hold here. Assumption (1) indicates that the encoded feature${\text{z}}_{i}$contains all the information from${\text{A}}_{i}$needed to predict$y_{i}$, and the residuals are independent of each other. Intuitively, we assume the motion${\text{c}}_{i}$explains part of the variability in brain networks${\text{A}}_{i}$, but this part of variability in${\text{A}}_{i}$does not relate to$y_{i}$. Of course, we can modify our model to add${\text{c}}_{i}$into the prediction of$y_{i}$in the case that${\text{c}}_{i}$is not independent of$y_{i}$. But it will make the final results difficult to interpret since we are mostly concerned with the relationship between${\text{A}}_{i}$and$y_{i}$.

Under Assumptions (1) and (2), we can express the conditional likelihood of${\text{A}}_{i},y_{i}$given${\text{c}}_{i}$as

$$\begin{array}{l} \text{log}\;\;p_{\theta }\left({\text{A}}_{i},y_{i}|{\text{c}}_{i}\right)\geq E_{{\text{z}}_{i}∼q_{\phi }}\;\left[\text{log}\;p_{\theta }\left({\text{A}}_{i}|{\text{z}}_{i},{\text{c}}_{i}\right)+\text{log}\;\;p_{\theta }\left(y_{i}|{\text{z}}_{i}\right)\right]\\ \;\;\;\;\;\;\;\;\;\;\;\;\;\;\;\;\;\;\;\;\;\;\;\;\;\;\;-KL\left[q_{\phi }\left({\text{z}}_{i}|{\text{A}}_{i}\right)\;||\;p\left({\text{z}}_{i}\right)\right]. \end{array}$$ (8)

The derivation is based on Jensen’s inequality; seeAppendix B. Intuitively, the lower bound of$\text{log}\;p_{\theta }\left({\text{A}}_{i},y_{i}|{\text{c}}_{i}\right)$in (8) consists of three parts. The first term,$\text{log}\;p_{\theta }\left({\text{A}}_{i}|{\text{z}}_{i},{\text{c}}_{i}\right)$, is a reconstruction error that utilizes log-likelihood to measure how well the generative model in (3) reconstructs${\text{A}}_{i}$. The second term,$\text{log}p_{\theta }\left(y_{i}|{\text{z}}_{i}\right)$, measures the trait prediction accuracy. The third KL divergence term is a regularizer that pushes$q_{\phi }\left({\text{z}}_{i}|{\text{A}}_{i}\right)$to be close to its prior$N\left(\text{0},{\text{I}}_{K}\right)$so that we can sample${\text{z}}_{i}$easily.

For the second term in the objective function (7), we can upper bound$I\left({\text{z}}_{i},{\text{c}}_{i}\right)$as

$$\begin{array}{l} I\left({\text{z}}_{i},{\text{c}}_{i}\right)\;\leq E_{{\text{A}}_{i}}\left[KL\left[q_{\phi }\left({\text{z}}_{i}|{\text{A}}_{i}\right)\;\;||\;q_{\phi }\left({\text{z}}_{i}\right)\right]\right]\\ \;\;\;\;\;\;\;\;\;\;\;\;\;\;\;-E_{{\text{A}}_{i},{\text{c}}_{i},{\text{z}}_{i}∼q_{\phi }}\left[\text{log}\left({\text{A}}_{i}|{\text{z}}_{i},{\text{c}}_{i}\right)\right], \end{array}$$ (9)

which consists of two parts: the KL divergence between$q_{\phi }\left({\text{z}}_{i}|{\text{A}}_{i}\right)$and its marginal$q_{\phi }\left({\text{z}}_{i}\right)$to ensure less variability across the input data${\text{A}}_{i}$, and the reconstruction error. SeeAppendix Cfor detailed derivations of (9).

Combining the log-likelihood and the mutual information terms, our training objective in (7) can be expressed as

$$\begin{array}{l} ℒ\left({\text{A}}_{i},y_{i},{\text{c}}_{i};\theta ,\phi \right)=E_{{\text{A}}_{i},y_{i},{\text{c}}_{i}}\left[\text{log}\;p_{\theta }\left({\text{A}}_{i},y_{i}|{\text{c}}_{i}\right)\right]-\lambda I\left({\text{z}}_{i},{\text{c}}_{i}\right)\\ \;\;\;\;\geq -E_{{\text{A}}_{i},y_{i},{\text{c}}_{i}}\left[KL[q_{\phi }\left({\text{z}}_{i}|{\text{A}}_{i}\right)\;\|\;p\left({\text{z}}_{i}\right)\right]\\ \;\;\;\;\;\;\;+\lambda KL\left[q_{\phi }\left({\text{z}}_{i}|{\text{A}}_{i}\right)\;\|\;q_{\phi }\left({\text{z}}_{i}\right)\right]\\ \;\;\;\;-\left(1+\lambda \right)E_{{\text{z}}_{i}∼q_{\phi }}\left[\text{log}\;p_{\theta }\left({\text{A}}_{i}|{\text{z}}_{i},{\text{c}}_{i}\right)\right]+\text{log}\;p_{\theta }\left(y_{i}|{\text{z}}_{i}\right)]. \end{array}$$ (10)

Our goal is to maximize$ℒ\left({\text{A}}_{i},y_{i},{\text{c}}_{i};\theta ,\phi \right)$through its lower bound (10). In practice, the expectation in (10) is intractable and we employ Monte Carlo approximation for computation; seeAppendix D. We define the approximated objective as$\tilde{ℒ}\left({\text{A}}_{i},y_{i},{\text{c}}_{i};\theta ,\phi \right)$, and implement a stochastic variational BayesianAlgorithm 1to update the parameters using minibatch training. For the regression module, we consider$p_{\theta }\left(y_{i}|{\text{z}}_{i}\right)$as a univariate Gaussian, that is,$y_{i}∼N\left({\text{z}}_{i}^{⊤}\beta +b,\sigma^{2}\right)$, where$\beta ,b,\sigma^{2}\in \theta$are parameters to be learned. We list the model parameters and architecture in theAppendix E.

**Table:** 

**Algorithm 1.** Motion-Invariant Variational Autoencoders
**Input:** ${\left\{{\text{A}}_{i}\right\}}_{i=1}^{N},{\left\{y_{i}\right\}}_{i=1}^{N},{\left\{{\text{c}}_{i}\right\}}_{i=1}^{N}$ .
Initialize θ,ϕ
** while** not converged **do**
Sample a batch of ${\left\{{\text{A}}_{i}\right\}}_{i=1}^{m}$ , denoted as ${\text{A}}_{m}$ .
** for all** ${\text{A}}_{i}\in {\text{A}}_{m}$ **do**
Sample ${\#}_{i}\in N\left(0,{\text{I}}_{K}\right)$ .
Compute ${\text{z}}_{i}=\mu_{\phi }\left({\text{A}}_{i}\right)+{\#}_{i}⊙\Sigma_{\phi }\left({\text{A}}_{i}\right)$
where ⊙ denotes the inner product operator.
Compute the gradients $\nabla_{\theta }\tilde{ℒ}\left({\text{A}}_{i},y_{i},{\text{c}}_{i};\theta ,\phi \right)$
and $\nabla_{\phi }\tilde{ℒ}\left({\text{A}}_{i},y_{i},{\text{c}}_{i};\theta ,\phi \right)$ with ${\text{z}}_{i}$ .
** end for**
Average the gradients across the batch.
Update θ,ϕ using gradients of θ,ϕ .
** end while**
Return θ,ϕ .

## 4 Simulation Study

**Fig. 3.**
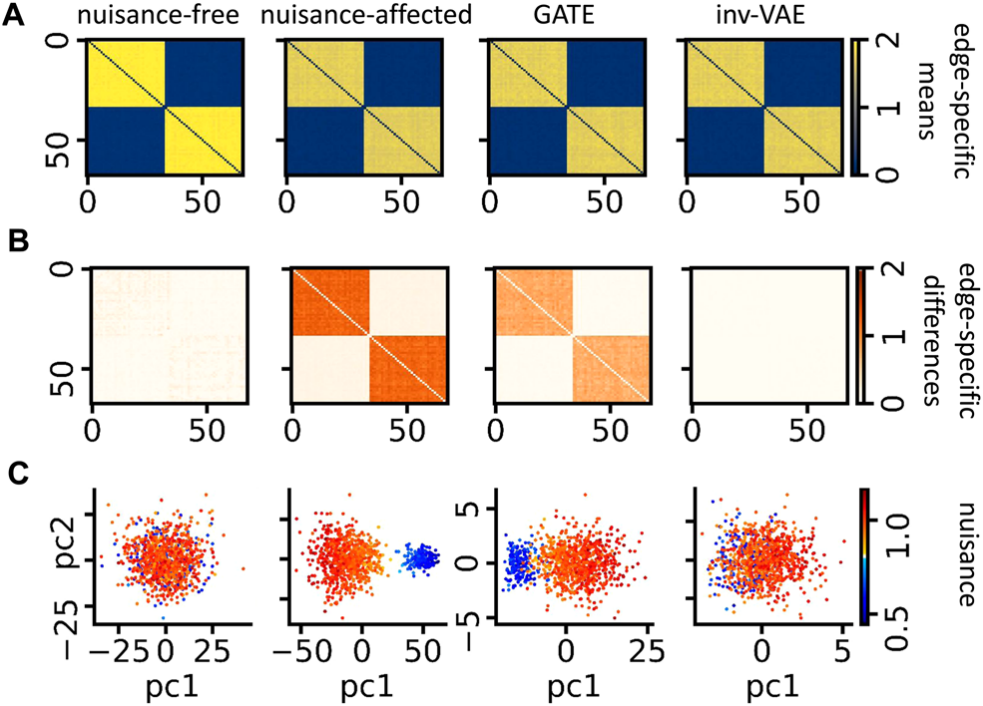
(A) Edge-specific means of simulated networks, nuisance-affected networks, reconstructed networks by GATE, and nuisance-corrected networks by inv-VAE. (B) Edge-specific differences between networks affected by large and small nuisance artifacts. The order of plots follows that in (A). (C) PCA projections of simulated networks, nuisance-affected networks, latent embeddings learned by GATE, and invariant embeddings from inv-VAE colored by the amount of nuisance artifacts.

We first conduct a simulation study to verify the efficacy of our method in removing unwanted information from the data. We simulate random graphs with the community structure (Nowicki & Snijders, 2001) using the Python package*NetworkX*. Random graphs of two communities are considered: Nodes in the same group are connected with a probability of 0.25, and nodes of different groups are connected with a probability of 0.01. We simulate 1,000 such networks${\left\{{\text{A}}_{i}\right\}}_{i=1}^{n}$, where${\text{A}}_{i}\in {\mathbb{R}}^{V\times V}$withV=68nodes andn=1,000.Figure 3(A)displays the average network across 1,000 simulated networks. Next, we simulate a*nuisance*variable,${\left\{s_{i}\right\}}_{i=1}^{n}$, by sampling20%of its elements fromN(0.6, 0.05)and the rest fromN(1, 0.01). We generate the nuisance-affected networks${\tilde{\text{A}}}_{i}$by propagating$s_{i}$across all edges in${\text{A}}_{i}$as follows:

$$\begin{array}{ccc} {\tilde{\text{A}}}_{i}={\left(s_{i}{\text{A}}_{i}\right)}^{⊤}\left(s_{i}{\text{A}}_{i}\right). & & \end{array}$$ (11)

Intuitively,$s_{i}∼N\left(0.6,\;0.05\right)$introduces large artifacts, since its multiplication with${\text{A}}_{i}$in (11) results in a${\tilde{\text{A}}}_{i}$with reduced edge values;$s_{i}∼N\left(1,\;0.01\right)$generates small artifacts as the multiplication operation in (11) changes the edges of${\text{A}}_{i}$to a lesser extent. The average network across 1,000 such nuisance-affected networks is displayed inFigure 3(A). To visualize the impact of the simulated nuisance variable, the edge-specific differences of the large and small nuisance groups are computed for both simulated networks${\left\{{\text{A}}_{i}\right\}}_{i=1}^{n}$and nuisance-affected networks${\left\{{\tilde{\text{A}}}_{i}\right\}}_{i=1}^{n}$(Fig. 3(B)). We observe apparent differences in edge values between the large and small nuisance groups of${\left\{{\tilde{\text{A}}}_{i}\right\}}_{i=1}^{n}$. We further apply principal component analysis (PCA) to both${\left\{{\text{A}}_{i}\right\}}_{i=1}^{n}$and${\left\{\;{\tilde{\text{A}}}_{\text{i}}\right\}}_{i=1}^{n}$, and visualize their projections onto the first two principal components (PCs) inFigure 3(C). The first two PCs of${\left\{{\text{A}}_{i}\right\}}_{i=1}^{n}$are indistinguishable. Note that these simulated networks are nuisance free, even though we color their projections with the simulated nuisance for visualization purposes. On the contrary, we observe an apparent separation between the large and small nuisance groups of${\left\{{\tilde{\text{A}}}_{\text{i}}\right\}}_{i=1}^{n}$after introducing unwanted associations between the nuisance variable and the simulated networks.

To demonstrate the effectiveness of our method in removing artifacts introduced by the nuisance variable, we further compare inv-VAE with the graph autoencoder (GATE) developed inM. Liu et al. (2021). GATE has the same generative model as inv-VAE (seeSection 3) except without adjusting for${\text{c}}_{i}$. Specifically, GATE assumes the following generative process for the brain network${\text{A}}_{i}$:

$$\begin{array}{c} A_{i\left[uv\right]}|{\text{z}}_{i}∼\text{Poisson}\left(\lambda_{i\left[uv\right]}\left({\text{z}}_{i}\right)\right),\;{\text{z}}_{i}∼N\left(\text{0},{\text{I}}_{K}\right),\\ \lambda_{i\left[uv\right]}\left({\text{z}}_{i}\right)=\text{exp}\left(\xi_{uv}+\psi_{uv}\left({\text{z}}_{i}\right)\right),\\ \psi_{uv}\left({\text{z}}_{i}\right)=\sum_{r=1}^{R}\alpha_{r}X_{ur}\left({\text{z}}_{i}\right)X_{vr}\left({\text{z}}_{i}\right). \end{array}$$ (12)

Moreover, GATE learns the latent vector${\text{z}}_{i}$using the following objective

$$\begin{array}{l} ℒ\left({\text{A}}_{i},y_{i};\theta ,\phi \right)=E_{{\text{z}}_{i}∼q_{\phi }}\left[\text{log}\;p_{\theta }\left(y_{i}|{\text{z}}_{i}\right)\right]\\ \;\;\;+E_{{\text{z}}_{i}∼q_{\phi }}\left[\text{log}\;p_{\theta }\left({\text{A}}_{i}|{\text{z}}_{i}\right)\right]-KL\left[q_{\phi }\left({\text{z}}_{i}|{\text{A}}_{i}\right)\;||\;p_{\theta }\left({\text{z}}_{i}\right)\right] \end{array}$$ (13)

instead of the motion-invariant objective in (10). We train GATE using${\left\{{\tilde{\text{A}}}_{i}\right\}}_{i=1}^{n}$to learn latent representations of nuisance-affected networks. The learned latents are used by the generative model in (12) to reconstruct networks inFigure 3(A).Figure 3(B) and (C)shows the reconstructed edge-specific differences between the large and small nuisance group, and the first two PCs of the learned latent variables, respectively.

Next, we train inv-VAE with${\left\{{\tilde{\text{A}}}_{i}\right\}}_{i=1}^{n}$to learn parametersϕandθfor the inference and generative model. The inference model then learns motion-invariant latent representations, from which the generative model produces nuisance-corrected networks (Fig. 3(A)) by setting$s_{i}=1$. As previously defined,$s_{i}\approx 1$corresponds to small nuisance artifacts, whereas a small$s_{i}$represents large nuisance artifacts. This is because multiplication of${\text{A}}_{i}$with a small$s_{i}$in (11) shrinks its edge values to a large degree, whereas multiplication with$s_{i}\approx 1$keeps the edge values roughly unchanged. Between-group edge-specific differences of the nuisance-corrected networks, and the first two PCs of invariant latents are shown inFigure 3(B, C).Figure 3(B, C)together suggests that GATE reconstructs the artifacts introduced by the nuisance variable, and its learned latent embeddings are affected by nuisance artifacts. On the contrary, such edge-specific differences between the large and small nuisance groups do not exist in the nuisance-corrected networks from inv-VAE, and projections of invariant embeddings of the large and small nuisance groups are indistinguishable. This observation implies that inv-VAE has the ability to remove unwanted information from the data.

## 5 Applications to the ABCD and HCP Data

### 5.1 Understanding and removing motion artifacts

A major motivation of our work is to understand how motion affects learning a low-dimensional representation of the structural connectome and to remove motion-induced artifacts from the connectome data. For this purpose, we apply our inv-VAE to the ABCD (8,646 subjects) and HCP data (1,065 subjects), and compare inv-VAE with its competitors on two tasks: (1) learning a latent connectome representation that is uninformative of motion and (2) generating a motion-adjusted connectome. This section focuses on the FSL eddy head motion. We provide an additional analysis inAppendix Gon addressing residual misalignment after eddy motion correction.

We first average the*motion-affected brain network*${\text{A}}_{i}$over all individuals in the study to obtain the*motion-affected edge-specific means*(seeSection 2.2) of the ABCD and HCP datasets, respectively. We call the resulting matrices as “motion-affected,” meaning no motion adjustment has been done. The first columns ofFigure 4(A, D)show the motion-affected edge-specific means in both datasets. We assign subjects into a small and a large motion group for each type of movement; SeeSection 2.2for the cutoff thresholds for the large and small motion groups in both datasets. The first columns ofFigure 4(B, E)show the*motion-affected edge-specific differences*, obtained by subtracting the motion-affected edge-specific means of the small motion group from those of the large motion group. For both datasets, we notice apparent fiber count differences between the two motion groups in almost all pairs of brain regions. Such brain connection differences due to motion artifacts are more prominent when we visualize the first two PCs of brain networks between the two groups. In the first columns ofFigure 4(C, F), each point represents an individual colored with the corresponding amount of motion. In the first column ofFigure 4(C), the PCA projections of the large and small motion subjects are closer compared with those in the first column ofFigure 4(F). This may be explained by the amount of motion-induced artifacts in the data: participants of the HCP study are young adults who may move less during data acquisition relative to the young adolescent ABCD participants.

**Fig. 4.**
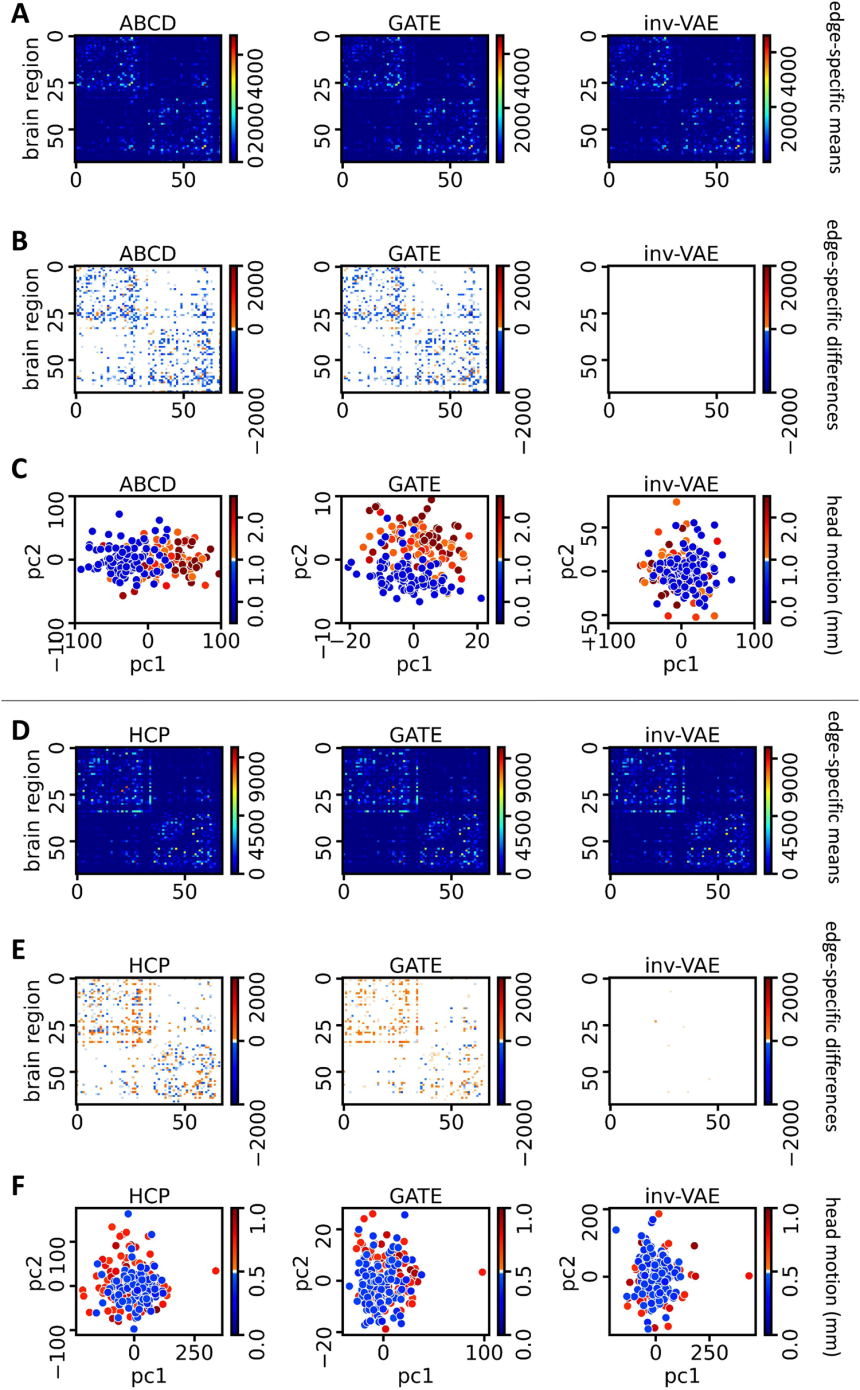
(A) From left to right: edge-specific means of ABCD brain networks, reconstructed brain networks by GATE, and motion-adjusted brain networks by inv-VAE, color coded according to fiber count. (B) From left to right: edge-specific differences (large group subtracts small group) for raw network data, GATE reconstructed network data, and inv-VAE reconstructed data, respectively, with color indicating differences in fiber count. (C) From left to right: the first two principal component scores of ABCD brain networks, latent embeddings learned by GATE, and invariant embeddings from inv-VAE for observations in the large- and small-motion groups, colored by the amount of head motion. (D)–(F) replicate the same content as (A)–(C) but emphasize head motion in the HCP data. Head motion is quantified in millimeters (mm).

Next, we apply GATE (M. Liu et al., 2021) to the ABCD and HCP data. For each dataset, GATE is trained using all brain networks${\text{A}}_{i}$’s to learn the model parametersθandϕ. Equipped with well-trained estimators, GATE obtains the learned latent variables from the inference model and reconstructs brain networks from the generative model in (12). We denote the*reconstructed brain network*as${\hat{\text{A}}}_{\text{i}}$. The second columns ofFigure 4(A, D)show the*reconstructed edge-specific means*averaged across all${\hat{\text{A}}}_{\text{i}}$’s in both datasets. In the second columns ofFigure 4(B, E),*reconstructed edge-specific differences*between the large and small motion group are shown. Although GATE can generate brain networks that resemble the observed data, motion artifacts are also undesirably reconstructed, as shown in the second columns ofFigure 4(B, E). The second columns ofFigure 4(C, F)show the first two PCs of the learned latents by GATE from the large and small motion groups. The gap between the two groups of latents suggests that representations learned by GATE are also corrupted by motion artifacts, which will further affect prediction and inferences.

For our inv-VAE, we use${\text{c}}_{i}\in \mathbb{R}$to represent thei-th subject’s head motion, with 0 representing the smallest amount of motion. To adjust for motion artifacts, we first train the inv-VAE model using the ABCD and HCP data separately. The inference model of inv-VAE learns a set of motion-invariant representations${\text{z}}_{i}$. Following the generative model inSection 3.1,${\text{z}}_{i}$are subsequently used to generate*motion-adjusted brain networks*, denoted as${\text{A}}_{i}^{*}$, by setting${\text{c}}_{i}$to 0 to remove motion artifacts. For each dataset, we average across all${\text{A}}_{i}^{*}$to obtain the*motion-adjusted edge-specific means*shown in the third columns ofFigure 4(A, D), which resemble the observed edge-specific means. The third columns ofFigure 4(B, E)show the*motion-adjusted edge-specific differences*by taking the difference between the motion-adjusted edge-specific means of the large and small motion groups. Compared with the color scale of the observed between-group edge-specific differences, the colors of the motion-adjusted between-group edge-specific differences are noticeably lighter, suggesting that brain connection differences between the large and small motion groups become smaller after motion adjustment. The third columns ofFigure 4(C, F)show the first two PCs of invariant representations${\text{z}}_{i}$for the ABCD and HCP data. The gap between large and small motion subjects is smaller after motion adjustment compared with the projections of the observed brain networks. Although the HCP subjects have smaller head movement and their connectomes are less affected by motion-induced artifacts, we still observe that motion adjustment minimizes the reconstructed structural connection difference between the large and small motion groups in the third column ofFigure 4(F).

Motion can significantly impact the acquired diffusion data, introducing signal loss, geometric distortion, misalignment volumes, and compromised diffusion measures. There have been many studies to see how motion impacts the diffusion MRI signal and constructed structural connectomes. For example,Andersson and Sotiropoulos (2016)highlight the susceptibility of diffusion MRI to artifacts caused by subject movements, resulting in both signal loss and geometric distortion. Additionally,Baum et al. (2018)demonstrate that motion has a noticeable impact on a considerable percentage of edges in structural brain networks. This effect is not limited to network edges but extends to significantly affected node strength and total network strength. In this work, we aim to understand how motion impacts our understanding of structural brain connectivity. For both ABCD and HCP studies, we first obtain the motion-affected edge-specific means via averaging${\text{A}}_{i}$’s across all individuals in the dataset. After training inv-VAE using all${\text{A}}_{i}$’s, we generate motion-adjusted networks${\text{A}}_{i}^{*}$’s from the invariant embeddings. We then average across all${\text{A}}_{i}^{*}$’s to construct the motion-adjusted edge-specific means. The differences between the motion-adjusted and motion-affected edge-specific means are shown inFigure 5(A, B). In both datasets, we observe more connections between the cingulate and the other brain regions in both hemispheres after motion adjustment, implying that brain connections related to the cingulate are more severely impacted by motion artifacts. There are also notable impacts in other regions, including the frontal, temporal, occipital, and parietal lobes, where we observe significant changes in connection strength after motion adjustment.

**Fig. 5.**
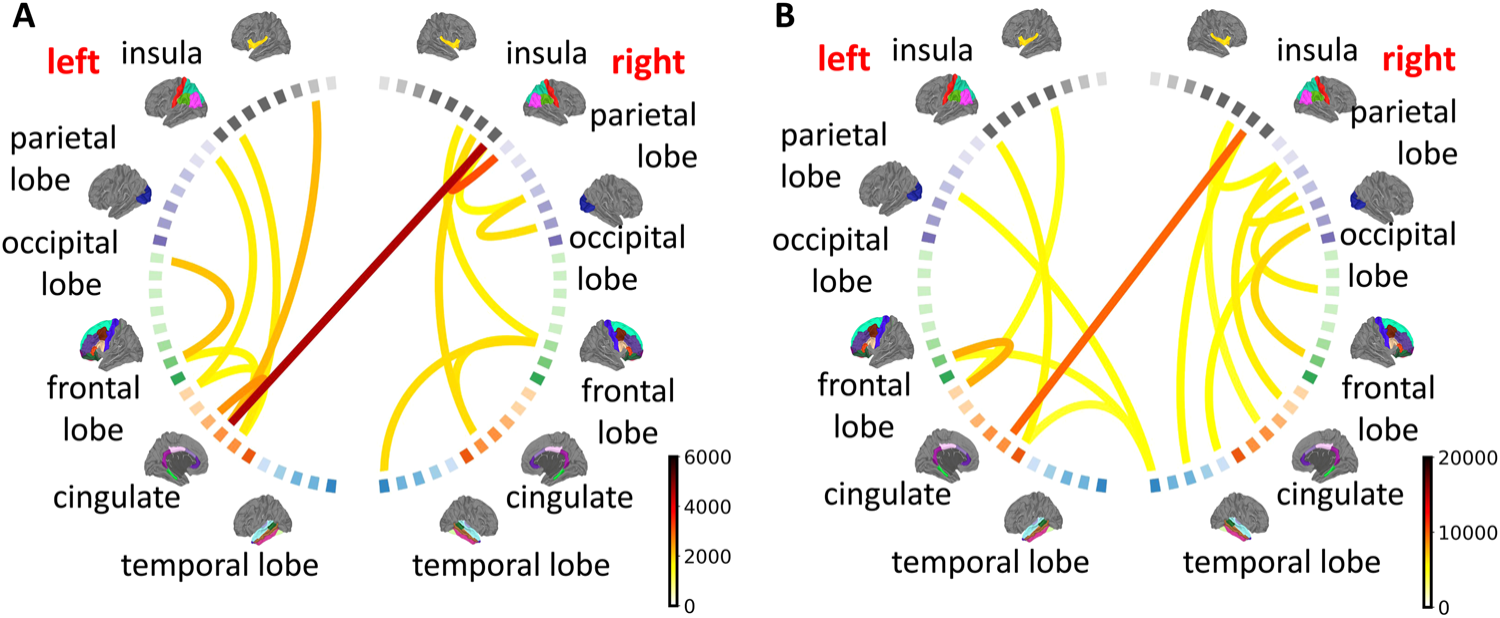
Structural connectivity differences between motion-adjusted and motion-affected ABCD (A) and HCP (B) brain networks; seeSection 5.1for definition. Each line connecting a pair of brain regions is colored by the corresponding fiber count difference after motion adjustment. We only show the 15 mostly changed brain connections.

### 5.2 Relating motion-adjusted connectomes to cognition-related traits

Our method can effectively adjust for motion-induced artifacts in the connectome data. A natural question is whether motion-invariant representations further our understanding of the relationship between brain connectomes and cognition-related traits. From the ABCD dataset, we extract the following cognitive traits as$y_{i}$: (1) picture vocabulary test score for language comprehension; (2) oral reading recognition test score for language decoding; (3) fluid composite score for new learning; (4) crystallized composite score for past learning; and (5) cognition total composite score for overall cognition capacity. From the HCP dataset, we consider similar traits: (1) picture vocabulary test score; (2) pattern completion test score for processing speed; (3) picture sequence test score for episodic memory; (4) list sorting test score for working memory; and (5) fluid intelligence score; seeGershon et al. (2013)for details about these traits. All traits are age corrected.

According toSection 3.2, we assume that motion$c_{i}$is independent of the trait of interest$y_{i}$. However, there might be significant correlations between clinically and scientifically interesting traits and subject motion. To mitigate the concern of erroneously attributing part of the connectome differences to motion rather than underlying traits, we preprocess trait$y_{i}$by regressing out$c_{i}$. For our analysis, we predict the residuals of$y_{i}$after regressing out$c_{i}$and examine the relationship between motion-adjusted connectomes and cognition-related traits.

To investigate whether our method outperforms other approaches in relating structural connectomes to traits, we compare inv-VAE with the following competitors: (1)*LR-PCA*(motion adjusted) that uses PCA to obtain a lower dimensional projection from each individual’s brain matrix, and then links$y_{i}$to the projection via a linear regression (LR). Translation and rotation are covariates that LR adjusts for; (2)*Sparse Orthogonal to Group (SOG)*(Aliverti et al., 2021) that utilizes matrix decomposition to produce an adjusted dataset that is statistically independent of the motion variable. We then regress$y_{i}$onto the obtained low-dimensional factorizations via LR; (3)*ComBat*(Johnson et al., 2007) that uses empirical Bayes for batch-effect removal, and returns a motion-adjusted dataset. We then apply PCA to the adjusted data to obtain low-dimensional projections as input to LR; (4)*GATE*(M. Liu et al., 2021) that has the same architecture as inv-VAE, but does not adjust for motion; seeSection 4for details about the model specification.

We use the above competing methods to produce 68-dimensional connectome representations for each individual, consistent with the recommended latent dimensionKin inv-VAE; seeSection 3. To assess trait prediction quality, we use the Pearson correlation coefficient to measure the correlation between the observed and predicted traits, and perform fivefold cross-validation (CV). A large correlation coefficient means the predicted trait scores more closely match the observed ones, indicating a stronger association between the low-dimensional representations of structural connectomes and the cognition-related traits.

A comparison of inv-VAE with the other approaches with respect to the trait-prediction quality is displayed inFigure 6, which shows that inv-VAE outperforms LR-PCA, ComBat, and SOG over all studied traits in both datasets, and either outperforms or is comparable with GATE over most selected traits. The prediction quality of SOG is comparable with GATE and inv-VAE for some cognitive traits: oral reading recognition and fluid intelligence score.

**Fig. 6.**
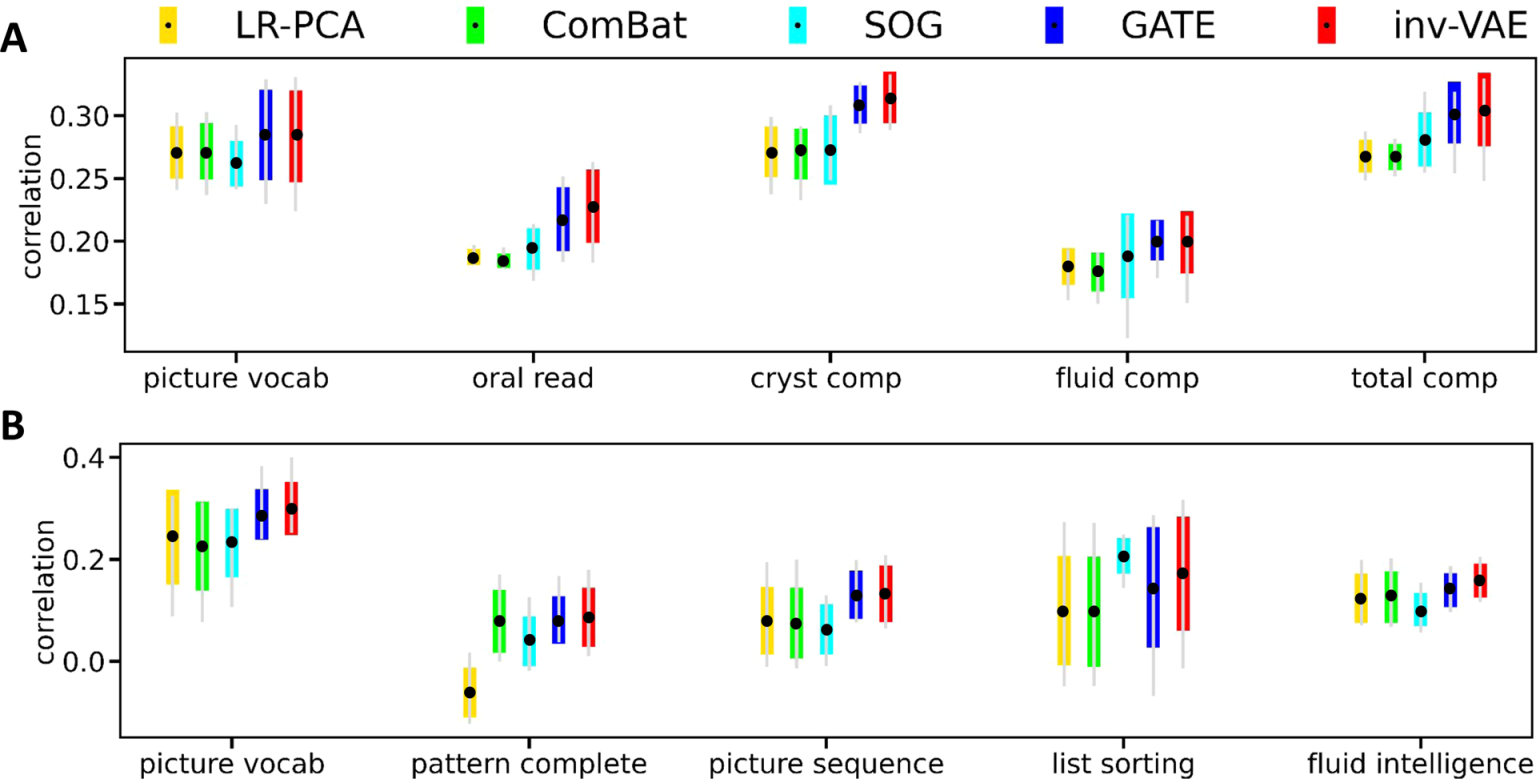
A comparison of all methods on trait prediction in the ABCD (A) and HCP (B) data. The error bars show the min, max, mean, and standard deviation of correlation coefficients from fivefold CV. We preprocess the target trait$y_{i}$by regressing out head motion. For each fold, we train inv-VAE on the training data. For the test data, we obtain trait predictions after setting$c_{i}$to 0 to remove head motion.

**Fig. 7.**
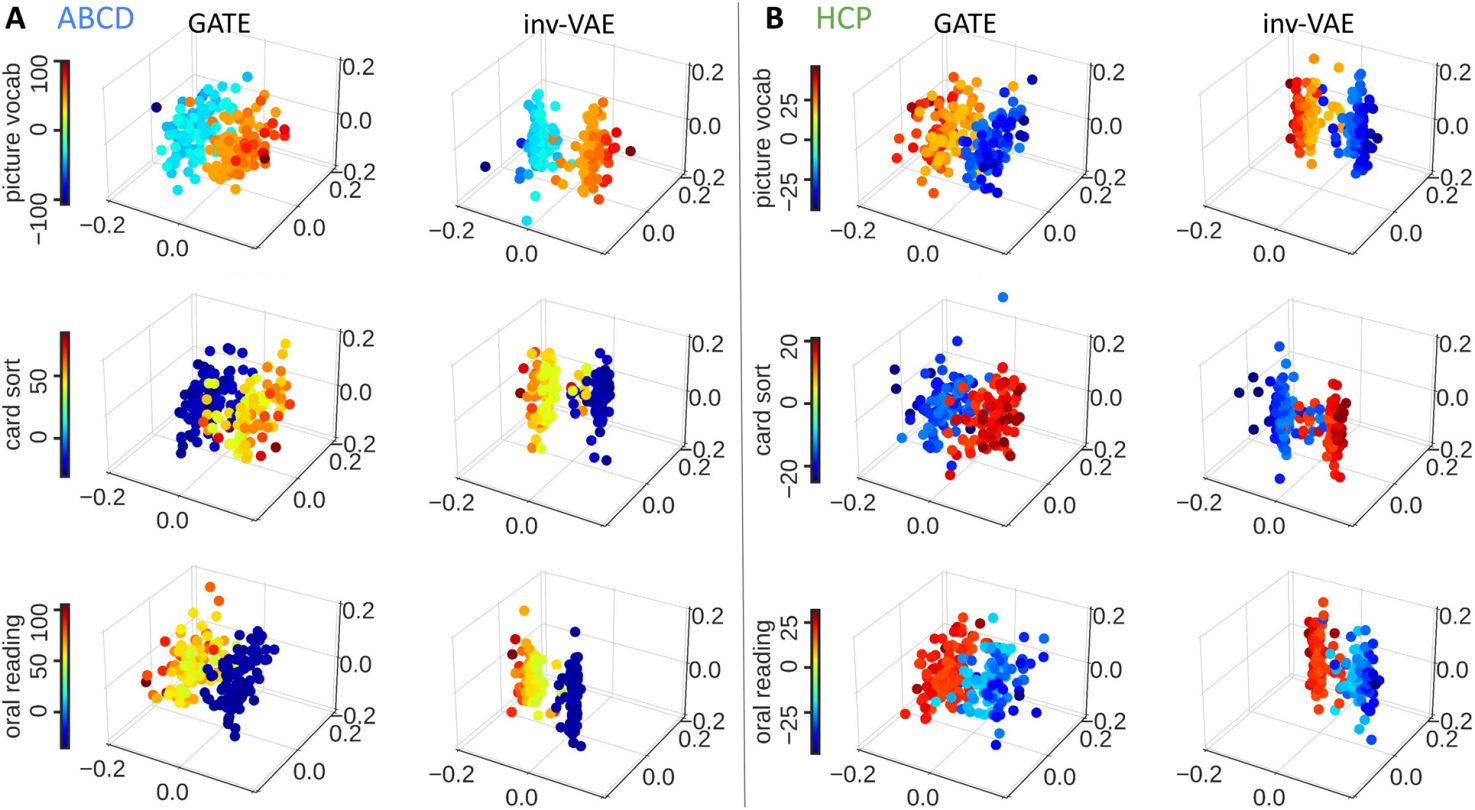
Relationships between learned embeddings and cognition-related traits in ABCD (A) and HCP (B) datasets. We preprocess the target trait$y_{i}$by regressing out head motion. Both inv-VAE and GATE are trained with brain networks of all individuals in each dataset to obtain the latent${\text{z}}_{i}$’s for the$y_{i}$’s. For each trait, latent features from 200 subjects are selected, with the first group of 100 having the lowest trait scores and the second group of 100 having the highest scores. The first three PCs of the posterior means of${\text{z}}_{i}$colored with their corresponding trait scores are displayed for each trait$y_{i}$. Latent embeddings produced by GATE and motion-invariant embeddings from inv-VAE are compared.

Another way to assess the predictive performance is to visualize the associations between low-dimensional representations of structural connectomes and cognition-related traits. Both inv-VAE and GATE produce latent features${\text{z}}_{i}$for each individual, which can be used for visualizations. Particularly, for the ABCD study, we consider picture vocabulary test, oral reading recognition test, and crystalized composite score; for the HCP study, we consider picture vocabulary test, oral reading recognition test, and dimensional change card sort test. Both inv-VAE and GATE are trained with brain networks of all individuals in each dataset to obtain the${\text{z}}_{i}$’s for the$y_{i}$’s. For each trait, latent features from 200 subjects are selected, with the first group of 100 having the lowest trait scores and the second group of 100 having the highest scores. Next, we visualize the first three PCs of the posterior means of${\text{z}}_{i}$colored with their corresponding trait scores inFigure 7. For both inv-VAE and GATE,Figure 7shows that representations from the two trait groups are separated, suggesting that structural connectomes are different among individuals with different cognitive abilities. We highlight that motion-invariant${\text{z}}_{i}$’s of the two groups are more separated than motion-affected${\text{z}}_{i}$’s from GATE, particularly for the oral reading recognition test. It implies that motion-invariant structural connectomes are more strongly associated with cognition-related traits than other approaches without motion adjustment.

## 6 Discussion

We develop a motion-invariant variational autoencoder (inv-VAE) for learning low-dimensional, motion-adjusted representations of structural connectomes. We apply inv-VAE to the Adolescent Brain Cognitive Development (ABCD) and Human Connectome Project (HCP) data and discover noticeable motion artifacts in both, despite the incorporation of motion correction procedures in preprocessing. This observation reinforces the need for effective motion mitigation strategies in connectome analysis. Being invariant to motion artifacts during the connectome modeling phase, inv-VAE shows improved performance in understanding the correlation between structural connectomes and cognition-related traits. While our inv-VAE is motivated to handle the motion confounder, it can be easily extended to handle other confounders, such as site batch effect and variation due to machine settings. Compared with popular batch effect removing models such as ComBat, the advantages of our inv-VAE are that (1) it can handle nonlinear objects (brain network data) and (2) it can deal with nonlinear batch effects. Therefore, we believe that our inv-VAE framework can be extended to broader brain imaging analysis tasks, highlighting the importance of confounder or batch effect adjustment methodologies.

Note that inv-VAE is developed under a generative model framework, which allows us to simulate brain networks under various conditions. The simulation of brain networks is crucial since brain network data are generally not widely available, and the extraction of brain networks from raw imaging data is far from straightforward. Our inv-VAE enables us to simulate$\left(A_{i},c_{i},y_{i}\right)$—that is, the brain network, undesirable artifacts, and cognition ability measures—simultaneously. Such simulated data can be freely shared with biostatisticians and data scientists who wish to develop statistical models for brain networks, but prefer not to get involved in the intricate process of brain imaging preprocessing.

Our method can model the spatially heterogeneous impact of motion: edges that share the same node can have correlations under our model since they are functions of both$z_{i}$and$c_{i}$. In this work, we simplify$c_{i}$to encode only a summary of subject-level motion artifacts. One possible future direction is to explore the representations of motion that can improve our generative model.

Our proposed inv-VAE currently models the motion-affected structural connectome using a Poisson generative model in conjunction with a GCN. Future work could incorporate pre-existing neuroscience knowledge regarding the effects of motion-induced artifacts on specific brain regions or connections to refine our model and improve the reconstruction of motion-adjusted structural connectomes. This could be achieved by leveraging empirical evidence, neuroscientific findings, or theoretical frameworks that shed light on the disparate impacts of motion on individual brain regions or connections. Through this integration of knowledge, we aim to boost the precision and accuracy of our motion artifact mitigation process, enabling us to better capture the subtle influences of motion on brain connectivity.

Another future direction is to leverage recent developments of generative modeling frameworks, such as variational diffusion models (D. Kingma et al., 2021), score-based generative models (Song & Ermon, 2020), and Hierarchical Variational Autoencoders (HVAE) (Sønderby et al., 2016). These models can be understood under the general Evidence Lower Bound (ELBO) method used here in this paper but with different constraints in the latent space (Luo, 2022). Moreover, we use the mean motion estimate across frames, as computed by the FSL eddy tool, to represent motion during diffusion MRI acquisition and ignore the longitudinal or higher order information in the motion. This simplification may not fully represent the impacts of motion on diffusion MRI data, and thus the motion-adjusted connectomes from our inv-VAE may not be completely invariant to motion. Future developments may incorporate more complex summaries of dynamic motion.

## Figures and Tables

**Fig. A1.**
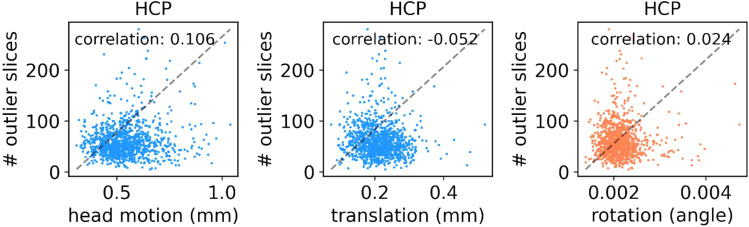
Pearson’s correlation between head motion, residual misalignment (translation and rotation), and outlier measure in the HCP dataset. Head motion and residual translation are measured in millimeters (mm), while residual rotation is measured in angles.

**Fig. A2.**
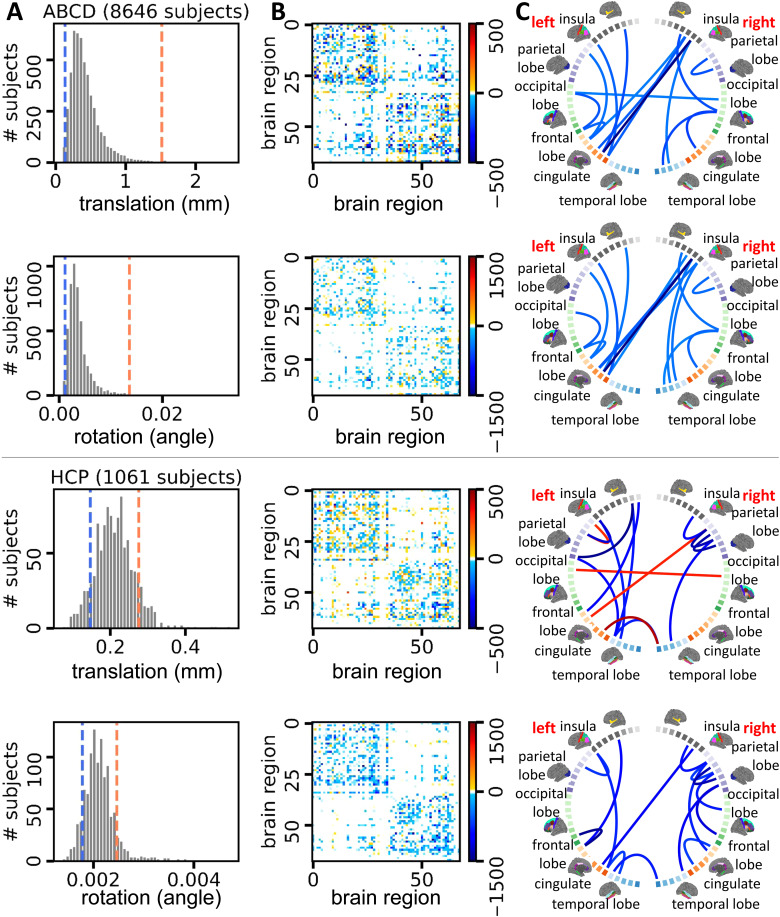
(A) Distributions of residual translation in millimeters (mm) and residual rotation in angles for the ABCD and HCP datasets. In ABCD data, the 5-th and 95-th percentiles are marked by the blue and orange lines. In HCP data, the blue and orange lines indicate the 10-th and 90-th percentiles. Subjects whose residual translation (rotation) falls below the blue line are assigned to the small translation (rotation) group, and those falling above the orange line are in the large translation (rotation) group. (B) Differences of mean networks between the large and small (large−small) translation (rotation) groups. (C) Plotting column (B) in circular plots. The color scale is the same as that in (B), but we only show 15 edges with the largest fiber count differences for display.

**Fig. A3.**
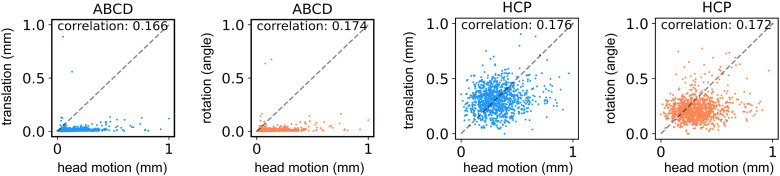
Pearson’s correlation between head motion and the residual translation and rotation in each dataset. Head motion is quantified in millimeters (mm) by computing the average displacement of each intracerebral voxel, then calculating the square root of the average squared displacements. Residual translation is measured in millimeters, while residual rotation is measured in angles.

**Fig. A4.**
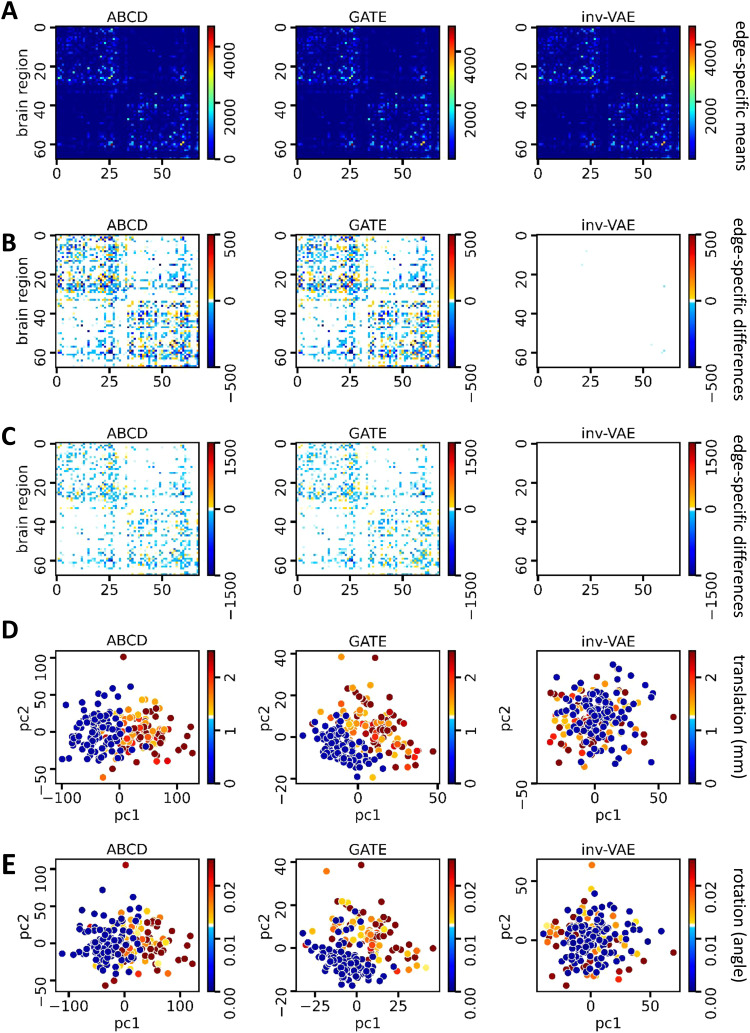
(A) From left to right: observed edge-specific means of ABCD brain networks, reconstructed brain networks by GATE, and residual-adjusted brain networks by inv-VAE, color coded by fiber count. (B) and (C): edge-specific differences (large group subtract small group) for residual translation and rotation, respectively, colored by differences in fiber count. Ordering follows that in (A). (D) and (E): from left to right—the first two PC scores of ABCD brain networks, latent embeddings learned by GATE, and invariant embeddings from inv-VAE for observations in the large and small translation (rotation) groups, colored by the amount of residual translation (D) and rotation (E), respectively. Residual translation is in millimeters (mm) and residual rotation is in angles.

**Fig. A5.**
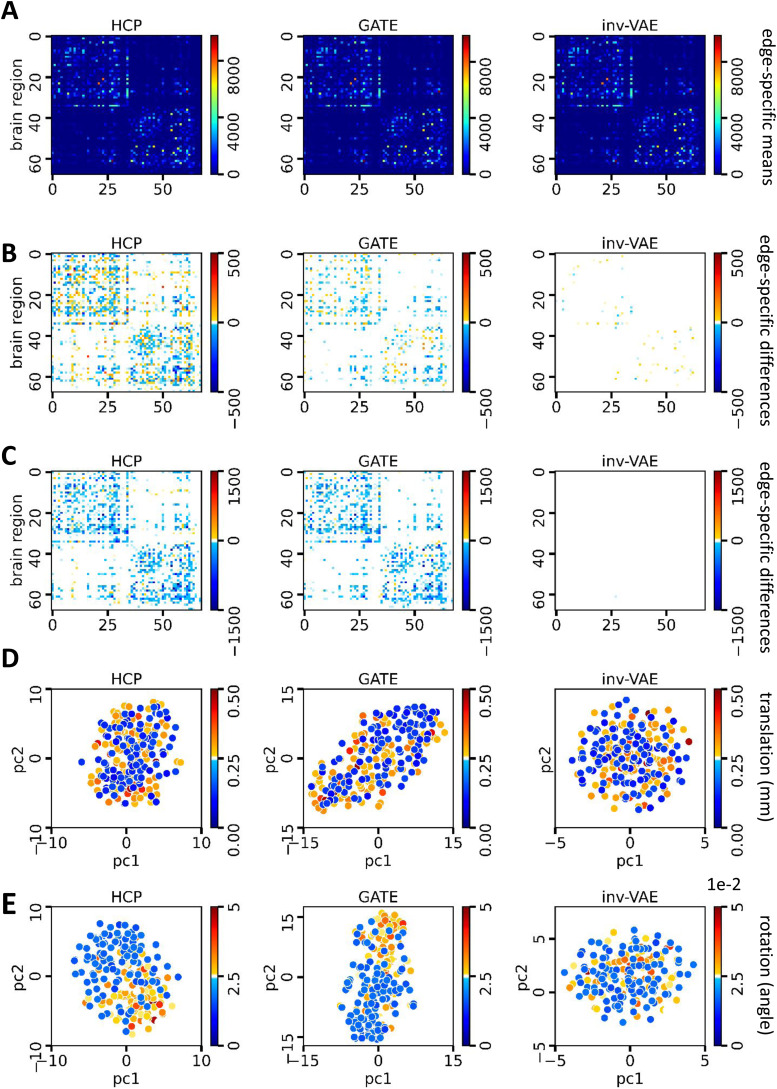
(A) From left to right: observed edge-specific means of HCP brain networks, reconstructed brain networks by GATE, and residual-adjusted brain networks by inv-VAE, color coded by fiber count. (B) and (C): edge-specific differences (large group subtract small group) for residual translation and rotation, respectively, colored by differences in fiber count. Ordering follows that in (A). (D) and (E): from left to right—the first two PC scores of HCP brain networks, latent embeddings learned by GATE, and invariant embeddings from inv-VAE for observations in the large and small translation (rotation) groups, colored by the amount of residual translation (D) and rotation (E), respectively. Residual translation is in millimeters (mm) and residual rotation is in angles.

**Fig. A6.**
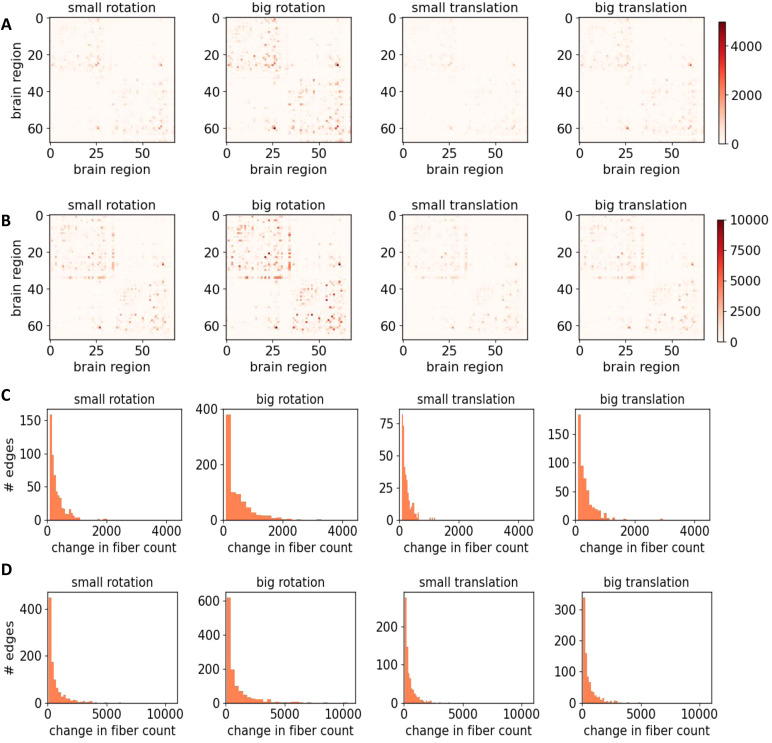
When setting$c_{i}=0$and applying the generative model, we observe small differences in the connectomes for the population with low residual artifacts, whereas large differences are observed in the connectomes of the populations with high residual artifacts. Edge-specific differences between the residual-adjusted networks and the original ABCD (A) and HCP (B) networks are averaged across all subjects in the small and large residual translation (rotation) groups. Colors represent the change in fiber counts after residual misalignment correction. The histograms depicting the scale of correction in fiber counts in the ABCD (C) and HCP (D) data are also presented. To improve clarity in visualization, only brain connections with a change in fiber counts exceeding 100 are displayed.

**Fig. A7.**
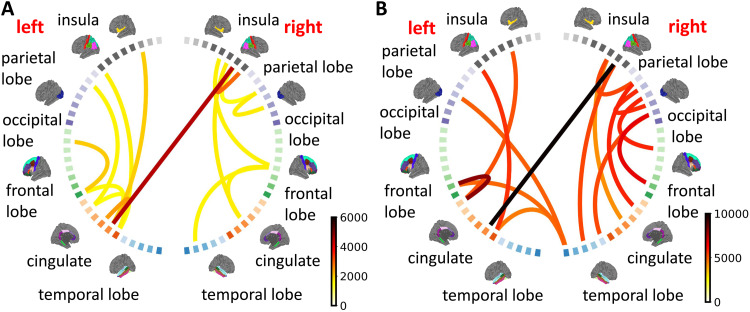
Structural connectivity differences between residual-adjusted and residual-affected ABCD (A) and HCP (B) brain networks. Each line connecting a pair of brain regions is colored by the corresponding fiber count difference after adjusting for residual misalignment. We only show the 15 mostly changed brain connections.

**Fig. A8.**
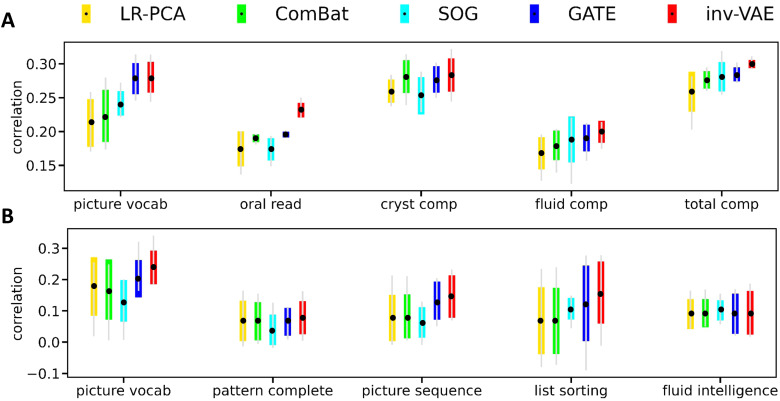
A comparison of all methods on trait prediction in the ABCD (A) and HCP (B) data. The error bars show the minimum, maximum, mean, and standard deviation of correlation coefficients from a fivefold CV. We preprocess the target trait$y_{i}$by regressing out residual misalignment. For each fold, we train inv-VAE on the training data. For the test data, we obtain trait predictions after setting$c_{i}$to 0 to remove residual misalignment.

**Fig. A9.**
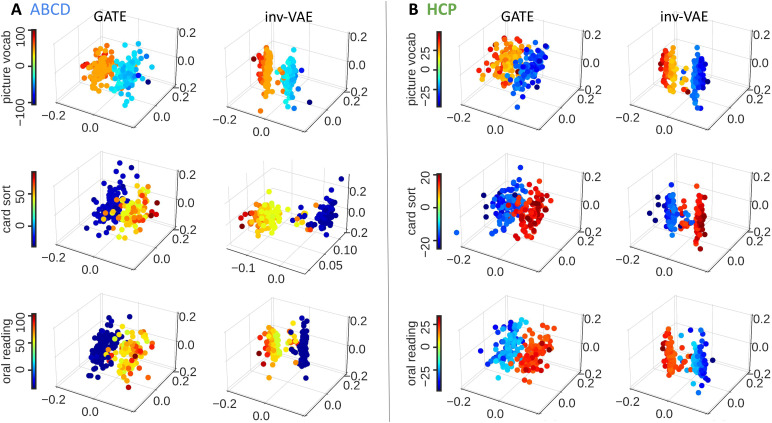
Relationships between learned embeddings and cognition-related traits in ABCD (A) and HCP (B) datasets. We preprocess the target trait$y_{i}$by regressing out residual misalignment. Both inv-VAE and GATE are trained with brain networks of all individuals in each dataset to obtain the latent${\text{z}}_{i}$’s for the$y_{i}$’s. For each trait, latent features from 200 subjects are selected, with the first group of 100 having the lowest trait scores and the second group of 100 having the highest scores. The first three PCs of the posterior means of${\text{z}}_{i}$colored with their corresponding trait scores are displayed for each trait$y_{i}$. Latent embeddings produced by GATE and residual-invariant embeddings from inv-VAE are compared.

**Fig. A10.**
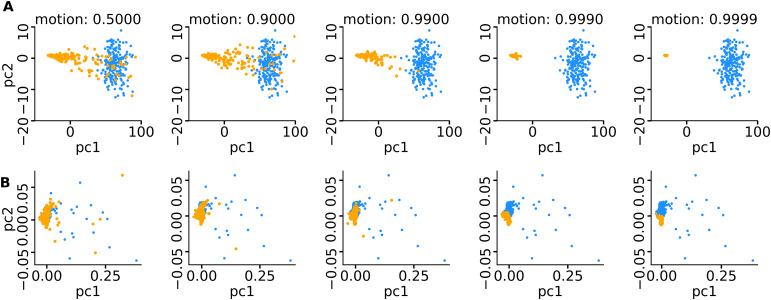
Analysis of the robustness of the proposed method under extreme motion and motion invariance test. (A) The first two PCs of the nuisance-free (blue) and the nuisance-affected (orange) networks under various levels of nuisances. (B) The first two PCs of the learned invariant latents for the nuisance-free (blue) and the nuisance-affected (orange) networks.

**Fig. A11.**
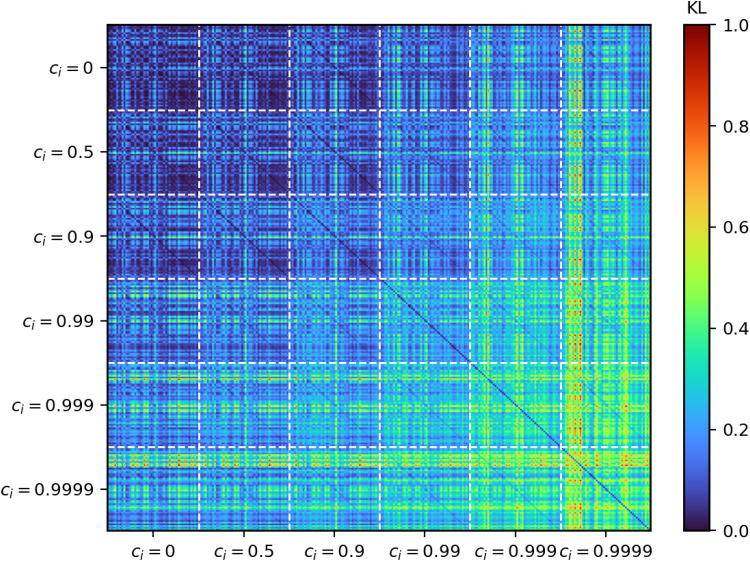
We construct a distance matrix to measure the KL divergence between each pair of$z_{i}$’s within and between the corresponding motion groups. Here,$c_{i}=0$denotes the simulated motion-free networks. We simulate motion-affected networks with five levels of motion$c_{i}=1-s_{i}\in \left\{0.5,0.9,0.99,0.999,0.9999\right\}$. Each motion group comprises 200 networks. Each matrix entry represents the KL divergence between a pair of networks, either within the same or different motion groups. The colors in the matrix reflect the normalized KL divergence.

## Data Availability

The data used in this study contain publicly available datasets from the Human Connectome Project^*^and the Adolescent Brain Cognitive Development Study^†^. The model training and testing code is available athttps://github.com/yzhang511/inv-vae^‡^.

## Appendix

This section provides model details and additional numerical studies. The appendix is organized as follows:

- Section A: Specification of GCN details used in both inv-VAE and GATE.
- Section B: Derivation of the joint likelihood of brain networks${\text{A}}_{i}$and cognition traits$y_{i}$conditional on the nuisance factor${\text{c}}_{i}$as defined inEquation (8).
- Section C: Derivation of the mutual information between latent variables${\text{z}}_{i}$and${\text{c}}_{i}$as defined inEquation (7).
- Section D and E: Implementation details, including Monte Carlo approximation of the training objective and model architecture details of our developed inv-VAE.
- Section F: Investigation of correlation between motion score and outlier measure to assess the relationship between head motion, residual misalignment, and outlier data.
- Section G: Analysis of the effect of residual misalignment adjustment on brain connectome reconstruction and trait prediction.
- Section H: Expanded simulation study to evaluate our method’s robustness in handling extreme motion-induced artifacts.
- Section I: Methods for extracting residual misregistration representations.

## Appendix A. GCN model specification

To exploit the local collaborative patterns among brain regions,M. Liu et al. (2021)propose to learn each region’s representation by propagating node-specifick-nearest neighbor information. We use the relative distance between a pair of brain regions to represent their locality. The relative distance is measured through the length of the white matter fiber tract connecting the pair, and stored in a matrix$\text{D}\in {\mathbb{R}}^{V\times V}$.$D_{uv}$denotes the fiber length between regionsuandv. Foru, itsk-nearest neighbors (kNNs) can be defined according toD. Graph convolution is defined to learn ther-th latent coordinate${\text{X}}_{r}({\tilde{\text{z}}}_{i})$for subjecti. In particular, anm-layer GCN is${\text{X}}_{r}^{\left(m\right)}\left({\tilde{\text{z}}}_{i}\right)=h_{m}\left({\text{W}}_{r}^{\left(m\right)}{\text{X}}_{r}^{\left(m-1\right)}\left({\tilde{\text{z}}}_{i}\right)+b_{m}\right),$for2≤m≤M, with${\text{X}}_{r}^{\left(1\right)}\left({\tilde{\text{z}}}_{i}\right)=h_{1}\left({\text{W}}_{r}^{\left(1\right)}{\tilde{\text{z}}}_{i}+b_{1}\right)$.${\text{X}}_{r}^{\left(m\right)}\left({\tilde{\text{z}}}_{i}\right)$denotes the output of them-th GCN layer,$h_{m}\left(\cdot \right)$is an activation function of them-th layer, and${\text{W}}_{r}^{\left(m\right)}$is a weight matrix characterizing the convolution operation at them-th layer. The embedding feature of each region at them-th layer is determined by the weighted sum of itself and its nearest neighbor regions at the(m−1)-th layer. Whenm=1,${\text{W}}_{r}^{\left(1\right)}\in {\mathbb{R}}^{V\times \left(K+C\right)}$maps${\tilde{\text{z}}}_{i}\in {\mathbb{R}}^{K+C}$to the latent space${\text{X}}_{r}^{\left(1\right)}\left({\tilde{\text{z}}}_{i}\right)\in {\mathbb{R}}^{V}$. Whenm≥2,${\text{W}}_{r}^{\left(m\right)}$is aV×Vmatrix with theu-th row${\text{w}}_{u\cdot }^{\left(r,m\right)}$satisfying$w_{uv}^{\left(r,m\right)}>0$ifv=uor$v\in k_{r}\text{NN}\left(u\right)$, and$w_{uv}^{\left(r,m\right)}=0$otherwise. Here,$k_{r}\text{NN}\left(u\right)$denotes theknearest neighbors of regionuin ther-th dimension of the latent space.

## Appendix B. Derivation of the joint likelihood of ${\text{A}}_{\text{i}},{\text{y}}_{\text{i}}$ conditional on ${\text{c}}_{\text{i}}$ in (8)

The joint likelihood of${\text{A}}_{i}$and$y_{i}$given${\text{c}}_{i}$is

$$\begin{array}{l} \begin{array}{cccc} \text{log}\;p_{\theta }\left({\text{A}}_{i},y_{i}|{\text{c}}_{i}\right) & =\text{log}\left(\int \frac{p_{\theta }\left({\text{A}}_{i},y_{i},{\text{z}}_{i}|{\text{c}}_{i}\right)}{q_{\phi }\left({\text{z}}_{i}|{\text{A}}_{i}\right)}q_{\phi }\left({\text{z}}_{i}|{\text{A}}_{i}\right)d{\text{z}}_{i}\right)=\text{log}\left(E_{z_{i}∼q_{\phi }}\left[\frac{p_{\theta }\left({\text{A}}_{i},y_{i},{\text{z}}_{i}|{\text{c}}_{i}\right)}{q_{\phi }\left({\text{z}}_{i}|{\text{A}}_{i}\right)}\right]\right). & & \end{array} \end{array}$$

By Jensen’s inequality,

$$\begin{array}{c} \text{log}\left(E_{z_{i}∼q_{\phi }}\left[\frac{p_{\theta }\left({\text{A}}_{i},y_{i},{\text{z}}_{i}|{\text{c}}_{i}\right)}{q_{\phi }\left({\text{z}}_{i}|{\text{A}}_{i}\right)}\right]\right)\geq E_{z_{i}∼q_{\phi }}\left[\text{log}\;p_{\theta }\left({\text{A}}_{i},y_{i},{\text{z}}_{i}|{\text{c}}_{i}\right)-\text{log}\;q_{\phi }\left({\text{z}}_{i}|{\text{A}}_{i}\right)\right]\\ =E_{z_{i}∼q_{\phi }}\left[\text{log}\;p_{\theta }\left({\text{A}}_{i},y_{i}|{\text{z}}_{i},{\text{c}}_{i}\right)+\text{log}\;p_{\theta }\left({\text{z}}_{i}|{\text{c}}_{i}\right)-\text{log}\;q_{\phi }\left({\text{z}}_{i}|{\text{A}}_{i}\right)\right]. \end{array}$$

Assuming that${\text{z}}_{i}$is independent of${\text{c}}_{i}$, we have$p_{\theta }\left({\text{z}}_{i}|{\text{c}}_{i}\right)=p\left({\text{z}}_{i}\right)$. We then have

$$\begin{array}{cccc} E_{z_{i}∼q_{\phi }}\left[\text{log}p_{\theta }\left({\text{A}}_{i},y_{i}|{\text{z}}_{i},{\text{c}}_{i}\right)\right] & +E_{z_{i}∼q_{\phi }}\left[\text{log}\;p_{\theta }\left({\text{z}}_{i}\right)-\text{log}\;q_{\phi }\left({\text{z}}_{i}|{\text{A}}_{i}\right)\right] & =E_{z_{i}∼q_{\phi }}\left[\text{log}\;p_{\theta }\left({\text{A}}_{i},y_{i}|{\text{z}}_{i},{\text{c}}_{i}\right)\right]-KL\left[q_{\phi }\left({\text{z}}_{i}|{\text{A}}_{i}\right)\;||\;p\left({\text{z}}_{i}\right)\right]. & \end{array}$$

## Appendix C. Derivation of mutual information $\text{I}\left({\text{z}}_{\text{i}},{\text{c}}_{\text{i}}\right)$ defined in (7)

From mutual information properties, we have that

$$\begin{array}{ccc} I\left({\text{z}}_{i},{\text{c}}_{i}\right)=I\left({\text{z}}_{i},{\text{A}}_{i}\right)-I\left({\text{z}}_{i},{\text{A}}_{i}|{\text{c}}_{i}\right)+I\left({\text{z}}_{i},{\text{c}}_{i}|{\text{A}}_{i}\right), & & \end{array}$$

where$I\left({\text{z}}_{i},{\text{c}}_{i}|\;{\text{A}}_{i}\right)=H\left({\text{z}}_{i}|{\text{A}}_{i}\right)-H\left({\text{z}}_{i}|\;{\text{A}}_{i},{\text{c}}_{i}\right)=H\left({\text{z}}_{i}|\;{\text{A}}_{i}\right)$$-H\left({\text{z}}_{i}|{\text{A}}_{i}\right)=0,$because the distribution of${\text{z}}_{i}$solely depends on${\text{A}}_{i}$not on${\text{c}}_{i}$. Using mutual information properties, we write$I\left({\text{z}}_{i},{\text{c}}_{i}\right)$as

$$\begin{array}{l} \begin{array}{cccc} I\left({\text{z}}_{i},{\text{c}}_{i}\right) & =I\left({\text{z}}_{i},{\text{A}}_{i}\right)-I\left({\text{z}}_{i},{\text{A}}_{i}|{\text{c}}_{i}\right) & =I\left({\text{z}}_{i},{\text{A}}_{i}\right)-H\left({\text{A}}_{i}|{\text{c}}_{i}\right)+H\left({\text{A}}_{i}|{\text{z}}_{i},{\text{c}}_{i}\right). & \end{array} \end{array}$$

Using variational inequality, we have

$$\begin{array}{l} I\left({\text{z}}_{i},{\text{c}}_{i}\right)\;\;\leq I\left({\text{z}}_{i},{\text{A}}_{i}\right)-H\left({\text{A}}_{i}|{\text{c}}_{i}\right)-E_{{\text{A}}_{i},{\text{z}}_{i},{\text{c}}_{i}∼q_{\phi }}[\log p_{\theta }\left({\text{A}}_{i}|{\text{z}}_{i},{\text{c}}_{i}\right)]\\ \;\;\;\;\;\;\;\;\;\;\;\;\;\;\;\;\;\;\;\;\;\;\;\;=E_{{\text{z}}_{i},{\text{A}}_{i}}\left[\log\;\;q_{\phi }\left({\text{z}}_{i}|{\text{A}}_{i}\right)-\log\;\;q_{\phi }\left({\text{z}}_{i}\right)\right]-H\left({\text{A}}_{i}|{\text{c}}_{i}\right)-E_{{\text{A}}_{i},{\text{c}}_{i},{\text{z}}_{i}∼q_{\phi }}\left[\log\;p_{\theta }\left({\text{A}}_{i}|{\text{z}}_{i},{\text{c}}_{i}\right)\right]\\ \;\;\;\;\;\;\;\;\;\;\;\;\;\;\;\;\;\;\;\;\;\;\;\;=E_{{\text{A}}_{i}}\left[KL\left[q_{\phi }\left({\text{z}}_{i}|{\text{A}}_{i}\right)||q_{\phi }\left({\text{z}}_{i}\right)\right]\right]-H\left({\text{A}}_{i}|{\text{c}}_{i}\right)-E_{{\text{A}}_{i},{\text{c}}_{i},{\text{z}}_{i}∼q_{\phi }}\left[\log\;p_{\theta }\left({\text{A}}_{i}|{\text{z}}_{i},{\text{c}}_{i}\right)\right], \end{array}$$

where$H\left({\text{A}}_{i}|{\text{c}}_{i}\right)$is a constant that can be ignored.

## Appendix D. Monte Carlo approximation of inv-VAE objective

We use Monte Carlo variational inference (D. P. Kingma, 2013) to approximate the intractable expectation in our invariant objective. Using reparametrization trick, for eachℓ=1,.. .,L, we sample${\text{z}}_{i}^{\ell }∼q_{\phi }\left({\text{z}}_{i}|{\text{A}}_{i}\right)=N(\mu_{\phi }\left({\text{A}}_{i}\right),$$\Sigma_{\phi }\left({\text{A}}_{i}\right))$, where$\mu_{\phi }\left({\text{A}}_{i}\right)\in {\mathbb{R}}^{K}$and$\Sigma_{\phi }\left({\text{A}}_{i}\right)\in {\mathbb{R}}^{K\times K}$. With${\text{z}}_{i}^{\ell }$, we decompose the objective in (10) into three parts: (1) The reconstruction error

$$\begin{array}{ccc} {ℒ}_{\text{recon}}=E_{{\text{z}}_{i}∼q_{\phi }}\left[\text{log}\;p_{\theta }\left({\text{A}}_{i}|{\text{z}}_{i},{\text{c}}_{i}\right)+\text{log}\;p_{\theta }\left(y_{i}|{\text{z}}_{i}\right)\right] & & \end{array}$$

can be approximated by${\tilde{ℒ}}_{\text{recon}}=\frac{1}{L}\sum_{\ell =1}^{L}(\text{log}\;p_{\theta }$$\left({\text{A}}_{i}|{\text{z}}_{i}^{\ell },{\text{c}}_{i}\right)+\;\text{log}\;p_{\theta }\left(y_{i}|{\text{z}}_{i}^{\ell }\right)).$(2) Because both$q_{\phi }\left({\text{z}}_{i}|{\text{A}}_{i}\right)$and$p_{\theta }\left({\text{z}}_{i}\right)$are normally distributed, the KL divergence between them,

$${ℒ}_{\text{prior}}=KL\left[q_{\phi }\left({\text{z}}_{i}|{\text{A}}_{i}\right)\;||\;p\left({\text{z}}_{i}\right)\right],$$

can be approximated by${\tilde{ℒ}}_{\text{prior}}=\frac{1}{2}\sum_{k=1}^{K}(\mu_{k}^{2}+\sigma_{k}^{2}-1$$-\text{log}{\left(\sigma_{k}\right)}^{2}),$where$\mu_{k},\sigma_{k}$are thek-th element of$\mu_{\phi }\left({\text{A}}_{i}\right)$and$\Sigma_{\phi }\left({\text{A}}_{i}\right)$. (3)$q_{\phi }\left({\text{z}}_{i}\right)$is the marginal distribution of$q_{\phi }\left({\text{z}}_{i}|{\text{A}}_{i}\right)$, and they are two Gaussians. We can approximate

$${ℒ}_{\text{marg}}=KL\left[q_{\phi }\left({\text{z}}_{i}|{\text{A}}_{i}\right)\;||\;q_{\phi }\left({\text{z}}_{i}\right)\right]$$

with the following pairwise Gaussian KL divergence (Louizos et al., 2015):

$$\begin{array}{l} {\tilde{ℒ}}_{\text{marg}}=KL\left[q_{\phi }\left({\text{z}}_{i}|{\text{A}}_{i}\right)\;\;||\;q_{\phi }\left({\text{z}}_{i}\right)\right]=\frac{1}{2}\left[\text{log}\frac{\text{det}\left(\Sigma_{0}\right)}{\text{det}\left(\Sigma_{1}\right)}+\text{tr}\left(\Sigma_{0}^{-1}\Sigma_{1}\right)-K+{\left(\mu_{0}-\mu_{1}\right)}^{⊤}\Sigma_{1}^{-1}\left(\mu_{0}-\mu_{1}\right)\right], \end{array}$$

where$\mu_{0}$and$\Sigma_{0}$parametrize$q_{\phi }\left({\text{z}}_{i}\right)$, and$\mu_{1}$and$\Sigma_{1}$parametrize$q_{\phi }\left({\text{z}}_{i}|{\text{A}}_{i}\right)$. The approximated invariant objective,$\tilde{ℒ}\left({\text{A}}_{i},y_{i},{\text{c}}_{i};\theta ,\phi \right)=\left(1+\lambda \right){\tilde{ℒ}}_{\text{recon}}-\lambda {\tilde{ℒ}}_{\text{marg}}-{\tilde{ℒ}}_{\text{prior}}$, is now differentiable with respect toθandϕ.

## Appendix E. Architecture of inv-VAE model

The architecture of our inv-VAE is presented inTable A1. Cross-validation is used to determine the best model for both the ABCD and HCP datasets. The latent dimension size and network depth are chosen to achieve the minimal training loss. We use the Adam optimizer (D. P. Kingma, 2014) to train both inv-VAE and GATE on a GPU device. During model training, the parameters inTable A1are set to be the same for both inv-VAE and GATE for a fair comparison. The learning rate is set to$5e^{-6}$in the simulation study and$2e^{-5}$in applications to the two datasets. For the minibatches, we choose a batch size of 32, and the batches are sampled uniformly at random at each epoch and repeated for 200 epochs in both simulation study and real applications.

**Table A1:** Model architecture of inv-VAE.

	Inference model (encoder)	Generative model (decoder)
inv-VAE	${\text{W}}_{1}\;:68\times 256$	*k* -NN: 32
	${\text{W}}_{2}\;:256\times 68$	K = 68
	$b_{1}\;:256\times 1$	*M* = 2
	$b_{2}\;:68\times 1$	*R* = 5
	$\varphi_{1}$ : ReLU	$h_{1}$ : Sigmoid
	$\varphi_{2}$ : Linear	$h_{2}$ : Sigmoid

*K*is the latent dimension of${\text{z}}_{\text{i}}$;*M*is the number of GCN layers;*R*is the latent dimension of$\text{X}({\text{z}}_{i});{\text{W}}_{.,}\;b_{.,}\;\varphi_{.}$are weights, biases, and activation functions in the encoder;h.are the activation functions in the decoder.

When the experiment’s objective is to adjust for motion-induced artifacts, as demonstrated inSection 5.1,90%subjects are included in the training set and10%subjects are included in the validation set to select optimal model parameters. When the goal is to predict cognition-related traits, as outlined inSection 5.2, we use fivefold cross-validation (CV) to designate 80% of the data as the training and validation data (in which, 90% are for training the model and 10% serve as the validation set to choose model parameters) and 20% as the test set for evaluating model predictions.

## Appendix F. Correlation between motion score and outlier measure

To clarify the specific aspects of image quality captured by our proposed motion measure, we conduct a comparison between the head motion estimate from the eddy tool, the residual misalignment (translation and rotation), and a summary of the outlier measure. InFigure A1, we examine the correlation between the proposed artifact measures and the number of outlier slices in the HCP dataset. The analysis reveals no significant relationship between outliers and either motion or residual misalignment, suggesting that the proposed measures capture other distinct artifacts present in the data.

## Appendix G. Inv-VAE with residual misalignment adjustment

In this section, we apply our proposed inv-VAE to residual misalignment adjustment. Residual misalignment refers to the remaining distortions and imperfections in image registration following FSL eddy correction. Our analyses of the ABCD and HCP datasets indicate that, although residual misalignment exhibits a low correlation with head motion obtained from FSL eddy (Fig. A3), it can still impact the constructed brain networks. Therefore, addressing this artifact is crucial as it enables us to correct for sources of artifacts that are not correlated with head motion.

To measure the residual misalignment, we rely onb=0images (images acquired without diffusion weighting). In the ABCD data, each individual has 7b=0frames evenly distributed throughout the diffusion scan, while each HCP subject has 18 frames withb=0. We use the FSL FLIRT tool to align otherb=0images to the firstb=0image with 6 degrees of freedom (DOF = 6, rigid body). Our analysis employs FSL version 5.0.9 for both the eddy and FLIRT tools. To quantify residual misalignment, we use the total amplitudes of rotation and translation. For each individual, we calculate the average translation and rotation displacements across all frames, providing a measure of residual misalignment. The detailed process for extracting these representations of residual translation and rotation is described inAppendix I.

The residual translation and rotation in the ABCD and HCP datasets demonstrate a relatively low correlation with the head motion (seeFig. A3), motivating us to check further the relationship between the residual misalignment and brain structural network. The results are displayed inFigure A2(A)–(C). In the column**A**ofFigure A2, we display the histograms of residual translation and rotation for ABCD and HCP subjects, with the blue and orange lines representing the 10th and 90th percentiles. Subjects with the residual translation (rotation) below the blue line are classified into the small translation (rotation) group, while those above the orange line belong to the large translation (rotation) group. In column (**B**), we show the mean network differences between the large and small translation (rotation) groups (large−small). Column (**C**) shows circular plots representing the data in panel (**C**), limited to the 15 edges showing the largest fiber count differences.

We further conduct a similar analysis parallel withSection 5.1“Understanding and Removing Motion Artifacts” to demonstrate the impact of residual misalignment and mitigate its effects on both the ABCD and HCP datasets. Firstly, we average the residual-affected brain network${\text{A}}_{i}$across all individuals in the study, obtaining the residual-affected edge-specific means for both datasets. The first columns ofFigures A4andA5(A)illustrate these residual-affected edge-specific means in both datasets. The first columns ofFigures A4andA5(B, C)display the differences in residual-affected edges in ABCD and HCP data, determined by subtracting the means of the small translation (rotation) group from those of the large translation (rotation) group. Additionally, we visualize the first two principal components (PCs) of brain networks in the first columns ofFigures A4andA5(D, E), with each point representing an individual colored according to their corresponding residual translation (rotation) displacement.

We then apply GATE (M. Liu et al., 2021) to both the ABCD and HCP datasets. The second columns ofFigures A4andA5(A)illustrate the reconstructed edge-specific means averaged across all reconstructed networks$\;{\hat{\text{A}}}_{\text{i}}$for both datasets. The second columns ofFigures A4andA5(B, C)show reconstructed edge-specific differences between the large and small translation (rotation) groups. The first two PCs of the learned GATE latents from the large and small translation (rotation) groups are displayed in the second columns ofFigures A4andA5(D, E)for residual translation (rotation).

To address artifacts due to residual misalignment, we train inv-VAE separately using the ABCD and HCP data. We generate residual-adjusted brain networks by setting${\text{c}}_{i}=\left(c_{i1},c_{i2}\right)$to 0, where$c_{i1}$and$c_{i2}$represent residual translational and rotational displacements, to eliminate residual artifacts. Averaging across all residual-adjusted networks for each dataset yields the residual-adjusted edge-specific means shown in the third columns ofFigures A4andA5(A). The third columns ofFigures A4andA5(B, C)illustrate the residual-adjusted edge-specific differences obtained by subtracting the residual-adjusted edge-specific means of the large and small translation (rotation) groups. The third columns ofFigures A4andA5(D, E)depict the first two PCs of invariant representations${\text{z}}_{i}$for both datasets. The gap between large and small translation (rotation) groups is reduced after residual adjustment compared with the projections of the observed brain networks.Figures A4andA5display the reconstructed fiber count differences between subject groups with low and high translation and rotation residual artifacts when*c_i_*= 0. To demonstrate how removing residual artifacts (by setting*c_i_*= 0) impacts the reconstructed brain connectomes across different population groups,Figure A6presents comparisons between residual-adjusted networks and original networks for each subject group, categorized by their varying levels of residual artifacts.

We further investigate how residual artifacts influence our understanding of structural brain connectivity. For both ABCD and HCP datasets, we first compute the residual-affected edge-specific means by averaging${\text{A}}_{i}$’s across all subjects in the dataset. After training inv-VAE using all${\text{A}}_{i}$’s, we generate residual-adjusted networks${\text{A}}_{i}^{*}$’s from the invariant embeddings. We then average across all${\text{A}}_{i}^{*}$’s to obtain the residual-adjusted edge-specific means.Figure A7(A,B)illustrates the differences between the residual-adjusted and residual-affected edge-specific means.

Parallel toSection 5.2, we explore whether residual-invariant representations can improve our understanding of the relationship between brain connectomes and cognition-related traits. To prevent mistakenly attributing connectome differences to residual misalignment rather than underlying traits, we predict the residuals of trait$y_{i}$after regressing out the residual artifacts,${\text{c}}_{i}=\left(c_{i1},c_{i2}\right)$, where$c_{i1}$and$c_{i2}$are the mean translational and rotational displacements across all frames.Figure A8compares inv-VAE’s trait-prediction quality with other baselines described inSection 5.2. The results demonstrate that inv-VAE outperforms LR-PCA, ComBat, and SOG across all studied traits in both datasets, while either surpassing or matching GATE’s performance for most selected traits.

We also assess predictive performance by visualizing associations between latent representations of structural connectomes and cognitive traits. For both ABCD and HCP studies, we select specific traits and use inv-VAE and GATE to produce latent features${\text{z}}_{i}$for each subject. We then choose 200 subjects (100 with the lowest scores, 100 with the highest) for each trait and visualize the first three PCs of the posterior means of their${\text{z}}_{i}$, colored by trait scores (Fig. A9). These plots show separation between high- and low-trait groups, indicating structural connectome differences related to cognitive abilities. Notably, inv-VAE’s residual-invariant${\text{z}}_{i}$’s show clearer separation than GATE’s residual-affected${\text{z}}_{i}$’s, especially for the oral reading recognition test. This suggests residual-invariant structural connectomes have stronger associations with cognitive traits compared with approaches without residual misalignment adjustment.

## Appendix H. Extreme motion and breakdown point

Given that HCP and ABCD data undergo multiple curation steps to exclude instances of very high motion, the threshold at which our proposed method might encounter limitations and fail to correct for extreme subject motion remains unclear. To address this, we conduct tests on our motion-invariant method using simulated data with varying levels of nuisance variables, evaluating the method’s robustness.

We expand the simulation study outlined inSection 4with varying motion levels. In particular, we simulate networks${\left\{{\text{A}}_{i}\right\}}_{i=1}^{n}$, where${\text{A}}_{i}\in {\mathbb{R}}^{V\times V}$withV=68nodes andn=200. We then introduce a nuisance variable,${\left\{s_{i}\right\}}_{i=1}^{n}$, by sampling its elements from$N\left(\mu_{j},\mu_{j}\right)$with$\mu_{j}\in \left\{0.5,\;0.1,\;0.01,\;0.001,\;0.0001\right\}$. To keep the notation consistent, we use$c_{i}$to denote the simulated motion, and set$c_{i}=1-s_{i}$. Motion-affected networks${\tilde{\text{A}}}_{i}$are generated by propagating$c_{i}$across all edges in${\text{A}}_{i}$using the expression:

$${\tilde{\text{A}}}_{i}={\left(\left(1-c_{i}\right){\text{A}}_{i}\right)}^{⊤}\left(\left(1-c_{i}\right){\text{A}}_{i}\right).$$
(14)

Intuitively, large$c_{i}$’s introduce large artifacts to the simulated networks, as the multiplication of$1-c_{i}$with${\text{A}}_{i}$inEquation (14)results in a${\tilde{\text{A}}}_{i}$with greatly reduced edge values; small$c_{i}$’s generate smaller artifacts as the multiplication operation inEquation (14)changes the edges of${\text{A}}_{i}$to a lesser extent.Figure A10(A)displays the first two principal components of the simulated brain networks colored by the motion group (motion free is in blue and motion affected is in orange), illustrating that as the simulated motion$c_{i}$increases, the distance between the two groups of networks becomes larger, making it more challenging for our proposed method to mitigate distortions from such artifacts.

We then train inv-VAE on the simulated motion-free and motion-affected networks to learn the parametersϕandθto learn the motion-invariant latent representations (seeFig. A10(B)). As defined earlier,$c_{i}\approx 0$corresponds to small motion artifacts, whereas large$c_{i}$’s represent large motion artifacts. If our method cannot accurately recover the motion-free$z_{i}$, the PCs of the invariant$z_{i}$’s from the motion-free and motion-affected group will be separated. InFigure A10(B), we visually display the invariant$z_{i}$’s of the two groups. Notably, when$c_{i}$is small, our proposed method performs well in recovering the motion-free latents. However, when$c_{i}$is large, our proposed method starts to break down, indicated by the clear separation between the two groups of latent representations.

We have conducted a simple motion-invariance test using both ABCD and HCP data. InFigures 4and5panels (D–E) in the manuscript, we present the first two principal component (PC) scores of observed brain networks, latent embeddings acquired through GATE, and invariant embeddings obtained from inv-VAE for brain networks in both large- and small-motion groups. Notably, we observe that both the PCs of the observed brain networks and GATE latents are correlated with the subject motion$c_{i}$. In contrast, the invariant$z_{i}$demonstrate independence from$c_{i}$.

Additionally, we conduct a simulation study to see whether the same subject, imaged with varying amounts of motion, would have the same$z_{i}$vector. For a given$A_{i}$, we can simulate various motion-affected networks${\tilde{\text{A}}}_{i}$with different motion parameters$c_{i}$. To check whether identical$z_{i}$can be obtained from these${\tilde{\text{A}}}_{i}$’s, we generate${\tilde{\text{A}}}_{i}$with a distinct$c_{i}=1-s_{i}$and examine whether the embeddings match those obtained when$c_{i}=0$. To test whether identical networks yield the same$z_{i}$under different motion levels, we use the symmetric Kullback–Leibler (KL) divergence to quantify the distance between the learned$z_{i}$’s. This choice is motivated by the fact that each$z_{i}$follows a multivariate normal distribution characterized by means and variances learned by our inference model:

$$\begin{array}{ccc} KL=\frac{1}{2}(D_{KL}(P||Q)+D_{KL}(Q||P)), & & \end{array}$$

wherePandQare the distributions of the motion-free$z_{i}$and motion-invariant$z_{i}$, respectively. If our method (inv-VAE) cannot accurately recover the same$z_{i}$, the network will have a large KL value.

In our simulation, we set$c_{i}=0$to simulate motion-free networks, and simulate motion-affected networks with five levels of motion$c_{i}=1-s_{i}\in \left\{0.5,\;0.9,\;0.99,\;0.999,\;0.9999\right\}$. Each motion group comprises 200 networks. We construct a distance matrix to measure the KL divergence between each pair of$z_{i}$’s within and between the corresponding motion groups. InFigure A11, each entry of the distance matrix represents the KL divergence between a pair of networks, either within the same or different motion groups. For visualization purposes, we normalize the KL divergence across network pairs within each submatrix inFigure A11. Examining the first row (column) of the distance matrix allows us to assess the motion invariance of$z_{i}$’s across various motion conditions—ranging from no motion ($c_{i}=0$) to increased levels of motion ($c_{i}\in \left\{0.5,\;0.9,\;0.99,\;0.999,\;0.9999\right\}$). Remarkably, when motion is not large, for example,$c_{i}\in \left\{0.5,\;0.9\right\}$, our proposed method successfully recovers the same$z_{i}$vector, evident from the distinct dark diagonal line in the submatrices in the first column (row) ofFigure A11(indicating low KL divergence, approximately 0). However, as motion levels ($c_{i}$) increase, our proposed method exhibits limitations, illustrated by the increased KL values between the motion-free$z_{i}$and the learned motion-invariant$z_{i}$.

## Appendix I. Representation of residual misregistration

For residual translation, it is represented as a 3D vector and we compute its amplitude using an$L_{2}$norm. For residual rotation, we utilize the method introduced inZhang, Descoteaux, et al. (2019). More specifically, let${\text{I}}_{3}$denote the identity element of rotation matricesSO(3). The tangent space at${\text{I}}_{3}$,$T_{I_{3}}\left(SO\left(3\right)\right)$forms a Lie algebra, which is usually denoted asso(3). The exponential map,exp:so(3)→SO(3), provides a mapping from the tangent space$T_{I_{3}}\left(SO\left(3\right)\right)$toSO(3). The inverse of the exponential map is called thelogmap.so(3)is a set of3×3skew-symmetric matrices. We use the following notation to denote any matrix${\text{P}}_{v}\in so\left(3\right)$:

$${\text{P}}_{v}=\left(\begin{array}{c} 0\\ v_{1}\\ -v_{2} \end{array}\begin{array}{c} -v_{1}\\ 0\\ v_{3} \end{array}\begin{array}{c} v_{2}\\ -v_{3}\\ 0 \end{array}\right),$$

where$v=\left[v_{1},v_{2},v_{3}\right]\in {\mathbb{R}}^{3}$. The exponential map forso(3)is given by Rodrigues’ formula,

$$\text{exp}\left({\text{P}}_{v}\right)=\{\begin{array}{cc} {\text{I}}_{3}, & \alpha =0\\ {\text{I}}_{3}+\frac{\text{sin}\left(\alpha \right)}{\alpha }{\text{P}}_{v}+\frac{1-cos\left(\alpha \right)}{\alpha^{2}}{\text{P}}_{v}^{\;\;2}, & \alpha \in \left(0,\pi \right), \end{array}$$

where$\alpha =\sqrt{\frac{1}{2}tr\left({\text{P}}_{v}^{\;\;T}{\text{P}}_{v}\right)}=∥v∥\in \left[0,\pi \right)$. Thelogmap for a matrixX∈SO(3)is a matrix inso(3), given by

$$\text{log}\left(\text{X}\right)=\{\begin{array}{cc} 0, & \alpha =0\\ \;\frac{\alpha }{2\text{sin}\left(\alpha \right)}\left(\text{X}-{\text{X}}^{T},\right) & \left|\alpha \right|\in \left(0,\pi \right), \end{array}$$

whereαsatisfiestr(X)=2cos(α)+1. Define a mappingΦto embed an element inso(3)to${\mathbb{R}}^{3}$,$\Phi :so\left(3\right)\to {ℜ}^{3}$,$\phi \left({\text{A}}_{v}\right)=v$. The vectorvis used in our paper to denote the residual rotation.

This is essentially an axis–angle representation of a rotation, which converts a rotation matrix into a compact, vector form that is easier to work with in many applications. To link this mapping to Euler angles, one would need to perform additional transformations, because Euler angles are a different way of representing 3D rotations. Euler angles specify three sequential rotations around the axes of a coordinate system, typically referred to as roll, pitch, and yaw.
